# Bio-assisted preparation of efficiently architectured nanostructures of γ-Fe_2_O_3_ as a molecular recognition platform for simultaneous detection of biomarkers

**DOI:** 10.1038/s41598-020-71934-7

**Published:** 2020-09-15

**Authors:** Sasikala Sundar, V. Ganesh

**Affiliations:** 1 Electrodics and Electrocatalysis (EEC) Division, CSIR – Central Electrochemical Research Institute (CSIR – CECRI), Karaikudi, Tamilnadu 630003 India; 2 Academy of Scientific and Innovative Research (AcSIR), Ghaziabad, 201002 India

**Keywords:** Materials science, Chemistry, Electrochemistry, Materials chemistry

## Abstract

Magnetic nanoparticles of iron oxide (γ-Fe_2_O_3_) have been prepared using bio-assisted method and their application in the field of biosensors is demonstrated. Particularly in this work, different nanostructures of γ-Fe_2_O_3_ namely nanospheres (NS), nanograsses (NG) and nanowires (NW) are prepared using a bio-surfactant namely Furostanol Saponin (FS) present in Fenugreek seeds extract through co-precipitation method by following “*green*” route. Three distinct morphologies of iron oxide nanostructures possessing the same crystal structure, magnetic properties, and varied size distribution are prepared and characterized. The resultant materials are analyzed using field emission scanning electron microscopy, transmission electron microscopy, powder X-ray diffraction, X-ray photoelectron spectroscopy, vibrating sample magnetometer and Fourier transform infrared spectroscopy. Moreover, the effect of reaction time and concentration of FS on the resultant morphologies of γ-Fe_2_O_3_ nanostructures are systematically investigated. Among different shapes, NWs and NSs of γ-Fe_2_O_3_ are found to exhibit better sensing behaviour for both the individual and simultaneous electrochemical detection of most popular biomarkers namely dopamine (DA) and uric acid (UA). Electrochemical studies reveal that γ-Fe_2_O_3_ NWs showed better sensing characteristics than γ-Fe_2_O_3_ NSs and NGs in terms of distinguishable voltammetric signals for DA and UA with enhanced oxidation current values. Differential pulse voltammetric studies exhibit linear dependence on DA and UA concentrations in the range of 0.15–75 µM and 5 μM – 0.15 mM respectively. The detection limit values for DA and UA are determined to be 150 nM and 5 µM. In addition γ-Fe_2_O_3_ NWs modified electrode showed higher sensitivity, reduced overpotential along with good selectivity towards the determination of DA and UA even in the presence of other common interferents. Thus the proposed biosensor electrode is very easy to fabricate, eco-friendly, cheaper and possesses higher surface area suggesting the unique structural patterns of γ-Fe_2_O_3_ nanostructures to be a promising candidate for electrochemical bio-sensing and biomedical applications.

## Introduction

Magnetic nanoparticles attract a great deal of interest among researchers across the globe owing to their unique magnetic properties such as superparamagnetic characteristics, low Curie temperature, negligible coercivity and high magnetic susceptibility that lead to applications in various fields^1,2^. Particularly, different forms of iron oxides such as magnetite (Fe_3_O_4_), maghemite (γ-Fe_2_O_3_), hematite (α-Fe_2_O_3_) and their corresponding nanoparticles are employed for many biological applications since they possess good biocompatibility. Among them, ferri-magnetic material namely, γ-Fe_2_O_3_ is very important for technological applications. Nanoscale γ-Fe_2_O_3_ has been used in diverse areas including high-density magnetic recording^3^, ferro-fluids^4^, drug delivery^5^, magnetic resonance imaging^6^, biosensors^7^, bio-probes^8^, hyperthermia treatment of cancer^9^, spintronics^10^, magneto-optics^11^, chemical catalysis^12^ and chemical sensing^13^ etc. Iron oxide nanoparticles also possess tunable magnetic properties depending upon their size^14,15^, shape^16–18^ and crystalline phase^19^.

Recent report suggests that on controlling the diameter and aspect ratio, nanowires (NWs) of γ-Fe_2_O_3_ were found to have tunable magnetic and optoelectronic properties^20^. For example, high-aspect ratio γ-Fe_2_O_3_ NWs found to possess a larger magnetic coercivity than the corresponding nanoparticles of same volume due to a shape-induced energy barrier which hinders the thermal rotation of magnetic moments^21^. Besides the shape, their crystallinity also has a profound effect on their magnetic and catalytic properties. It is reported that the rotation moment scales up against size of the energy barrier and the effect is directly correlated with the increasing crystallite volume^22^. For a given size of γ-Fe_2_O_3_ NWs, single crystal NW has the largest possible crystallite volume through which the energy barrier against thermal rotation moment is maximized. Therefore, it is highly desirable to synthesize single crystal γ-Fe_2_O_3_ NWs in order to take full advantage of their large aspect ratio, so that their coercivity values are enhanced for the specified applications. Conventionally, synthesis of γ-Fe_2_O_3_ NWs is carried out through the formation of intermediate NWs of other iron oxide phases (like α-Fe_2_O_3_, α-FeO(OH), or Fe_3_O_4_) using toxic chemicals followed by either reduction, oxidation or transformation processes. These kinds of procedures are usually lengthy, time consuming and difficult to accomplish a good control over the crystallinity and phase purity of the resultant nanostructures.

To overcome the above stated limitations, Furostanol Saponin (FS) present in the Fenugreek seeds extract is utilized to synthesize iron oxides of size and shape-controlled nanostructures along with control over their growth direction. For the first time, this proposed methodology has been used to produce high yield and high quality of γ-Fe_2_O_3_ nanograsses (NGs), nanowires (NWs) and nanospheres (NSs) via biogenic (*greener*) route^23,24^. The resultant γ-Fe_2_O_3_ nanostructures are characterized by using various spectroscopic and microscopic techniques. Further these materials are explored for the simultaneous electrochemical detection of neurotransmitters, the potential biomarkers of many diseases. Simultaneous electrochemical detection of analytes remains still a challenge and we have successfully demonstrated in this work the simultaneous electrochemical sensing of dopamine (DA) and uric acid (UA) using NWs and NSs of γ-Fe_2_O_3_. Such studies are performed by modifying glassy carbon electrode (GCE) with these materials that exhibit increased electrochemical sensing properties towards the detection of DA and UA. Although the basic materials are being self-assembled using chemicals that themselves are biologically unfriendly but the approach of directional self-assembly using a naturally available extract eliminates the need of some very toxic precursors and uneconomic lengthy processes that are otherwise indispensable for obtaining such directional self-assembly of nanomaterials.

## Results and discussion

### Morphological studies

**Figure 1:**
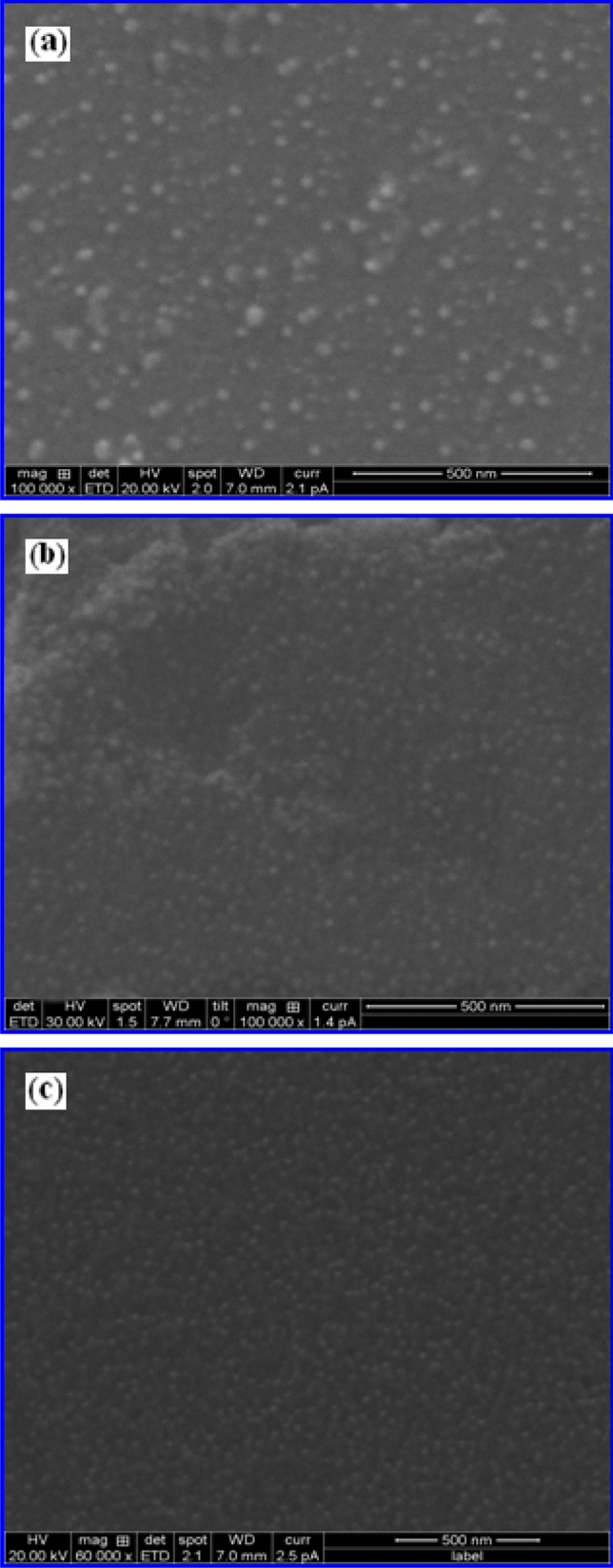
Morphological characteristics analyzed through FESEM images of γ-Fe_2_O_3_ nanostructures synthesized at 30 min using different volume % of FS namely, (**a**) 2% FS, (**b**) 4% FS and (**c**) 10% FS respectively.

In order to understand the formation process of different nanostructures of iron oxide synthesized using varied volume % of FS extracts, time-dependent evolution of these iron oxide nanostructures was analyzed by varying the time duration associated with the reaction. The samples were collected at different time intervals between 30 min and 3 h from the reaction mixture once the precipitate was formed and their corresponding FESEM images are shown in Fig. 1.

At the early stage of evolution process (30 min), it can be seen that the product is mainly composed of smaller spherical nanoparticles with an average particle size of about 10, 7 and 5 nm for 2%, 4% and 10% respectively. It is possible to obtain controlled sizes of spherical NSs of γ-Fe_2_O_3_ by controlling the volume % of FS extract (bio-surfactant) from 2 to 10% at a shorter reaction time of 30 min. When the concentration of FS was reduced from 10 to 2%, the size of the nanocrystals increased from 5 to 10 nm. It has been demonstrated that the spherical NSs are produced in all the volume % of FS (under the same reaction time of 30 min), indicating the homogeneity of the formation of NSs is increased with increasing concentration of FS. The reason is found to be, at a shorter period of reaction time (30 min) the dipolar attraction between these NSs is weaker and the steric repulsion is predominant. At this condition, only smaller particles of NSs are formed with almost no 1D nanostructures are generated.

**Figure 2:**
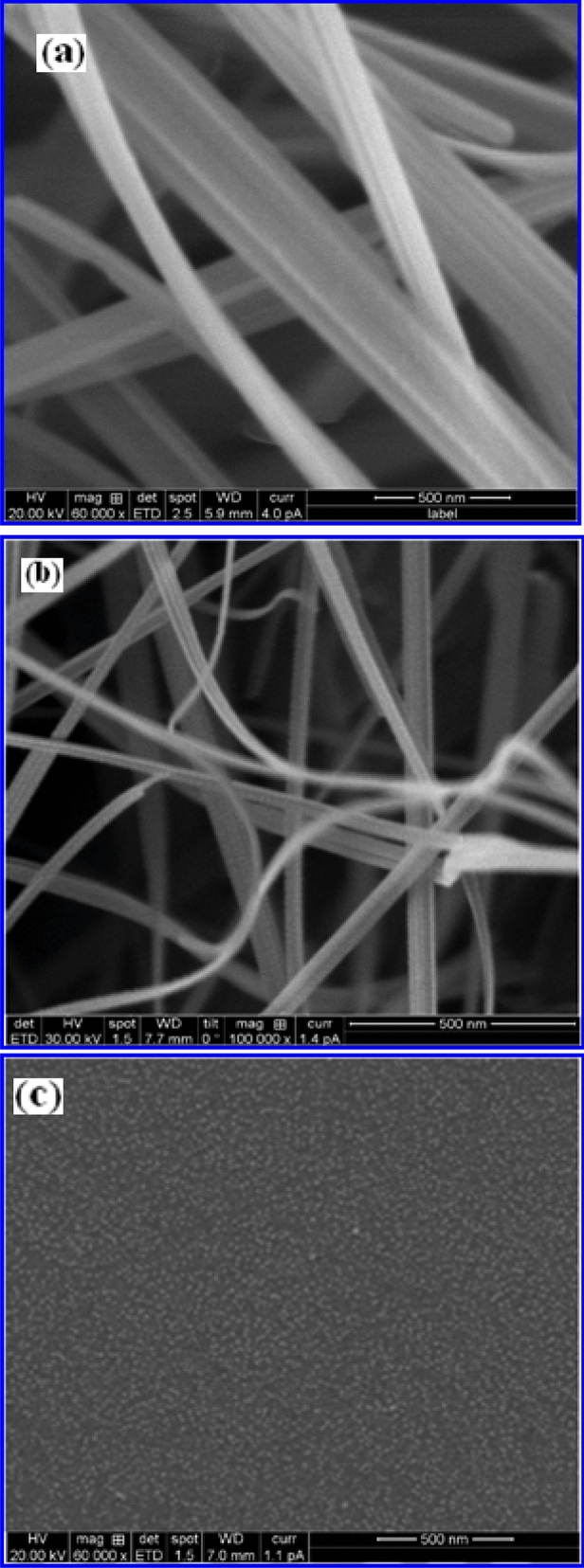
Structure and morphological characteristics analyzed using FESEM images of γ-Fe_2_O_3_ nanostructures synthesized at 3 h using different volume % of FS viz., (**a**) 2% FS, (**b**) 4% FS and (**c**) 10% FS respectively.

To investigate the effect of surfactant concentration and evolution time on the growth of 1D γ-Fe_2_O_3_ nanostructures, the ratio of iron precursors to FS concentration was varied at 2:1 ratio comprising of Fe and 2, 4, 10% of FS concentrations by allowing the reaction time of 3 h. Figure 2a–c show the corresponding FESEM images of γ-Fe_2_O_3_ grown at 2%, 4% and 10% concentrations for 3 h time duration. As the reaction time is increased, the spherical nanoparticles (observed for 30 min) are completely transformed to 1D nanostructures and found that the oriented aggregation has occurred when the reaction time is increased to 3 h.

From these images it is clear that the large number of γ-Fe_2_O_3_ NSs self-assembled into 1D nanostructures like NGs and NWs in case of 2% and 4% FS reaction medium respectively. At 2% FS concentration, γ-Fe_2_O_3_ NGs are formed with an average length and width of 2,500 nm and 100–300 nm. By increasing the concentration of FS from 2 to 4%, NWs are formed with an average length of 2000 nm and the diameter is determined to be 90 nm. The average width of these NWs is narrowed. It is interesting to note that the NW structure is formed when explicit lateral growth of NG is stopped and the longitudinal growth begins. These results revealed that the increased FS extract (more –OH ions) causing the end to close and width to decrease, and most of the saponin (–OH ions) acts as a shape controller to induce anisotropic growth. Therefore, –OH ions play a much crucial role in the formation of 1D nanostructure because of their stronger adsorption effect. Conversely, it can be seen that the obtained products are spherical in shape (monodispersed and non-agglomerated spherical nanoparticles) at higher concentration (10% FS) of surfactant.

The average particle size of these iron oxide nanospheres is found to be 12 nm. These results demonstrate that the FS concentration significantly influences the morphology of resultant iron oxide nanostructures. In case of spherical, crystalline γ-Fe_2_O_3_ NSs prepared using 10% FS, the lack of central symmetry due to the distribution of polar facets may result in dipolar attraction along the polar faces of these NSs. For γ-Fe_2_O_3_ nanocrystals, (311) plane is polarized which leads to dipolar attraction of γ-Fe_2_O_3_ nanoparticles along this axis^25,26^. It is well known that the dipole–dipole attraction favored the oriented attachment, but there is another steric or electrostatic repulsion which prevents them from aggregation. The steric repulsion originates from the stabilizing molecules (such as FS) on the surface of NSs, resulting in the repulsion of spherical particles within each other (10% FS). On the other hand, the stabilizing molecules maintain a dynamic equilibrium on the surface of these particles during the adsorption and desorption processes under certain conducive reaction conditions. Increasing the FS concentration in the reaction medium is more favourable for the formation of NSs and to prevent the formation of 1D nanostructures of γ-Fe_2_O_3_. Based on these results, it can be reasonably understood that 1D nanostructures are formed through the aggregation of NSs during the synthesis process, which is usually termed as “oriented attachment”. This mechanism involves a two-stage process including the formation of spherical nanoparticles and subsequently the aggregation of a string of these NSs^27^.

**Figure 3:**
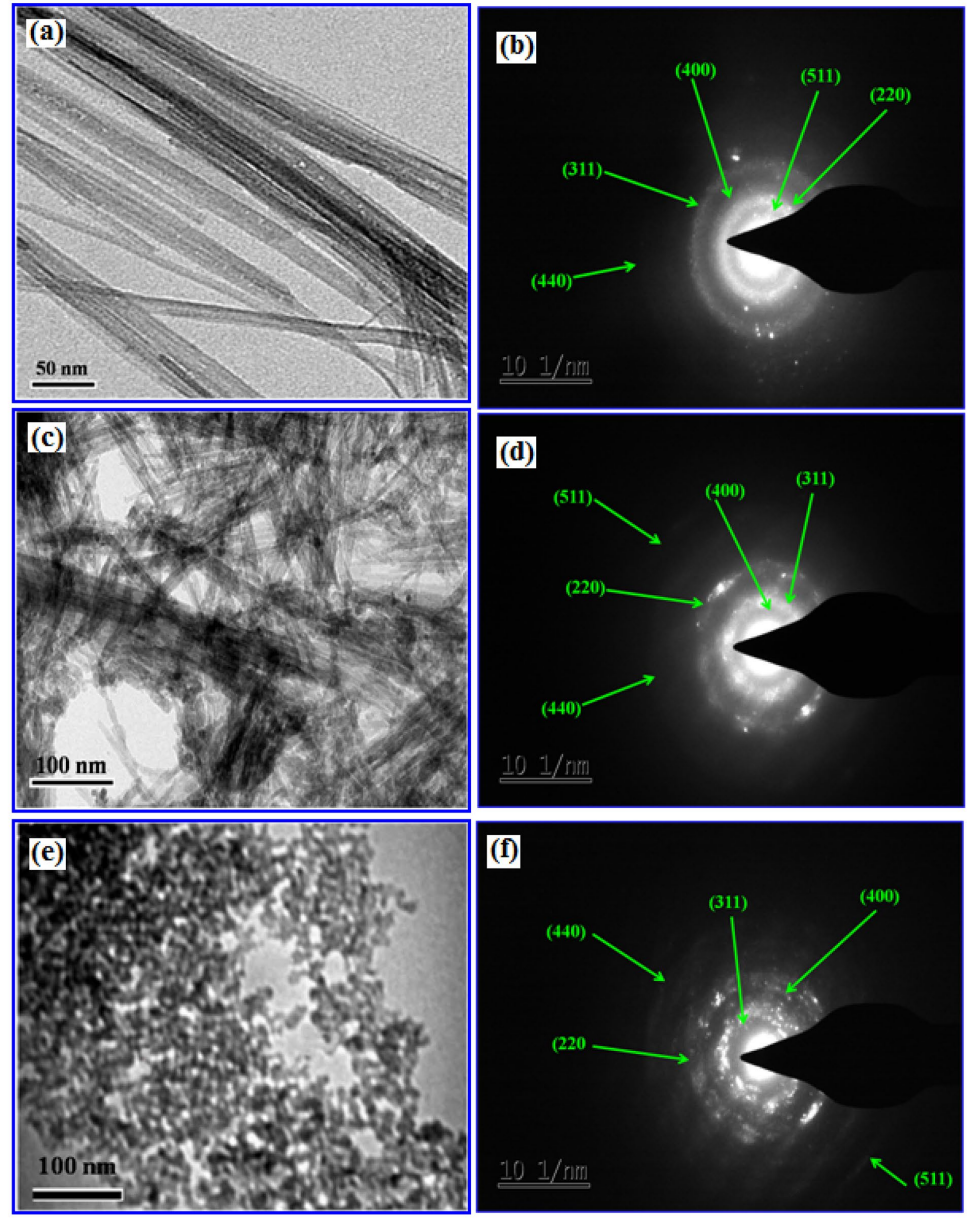
TEM images and selected area electron diffraction (SAED) patterns of γ-Fe_2_O_3_ nanostructures synthesized at 3 h using different volume % of FS viz., (**a**, **b**) 2% FS, (**c**, **d**) 4% FS, and (**e**, **f**) 10% FS respectively.

More detailed morphological analysis on the formation of γ-Fe_2_O_3_ nanostructures synthesized using various volume % of FS extract for 3 h time duration is illustrated by TEM images along with their corresponding selected area electron diffraction pattern shown in Fig. 3. These TEM images revealed that all the nanostructures synthesized using different % of FS concentrations are in different shapes but uniform in size. The NG and NW like morphology are observed for the iron oxide samples synthesized using 2% and 4% FS medium respectively. In contrast, for 10% FS concentration, non-agglomerated spherical nanoparticles are produced with homogenous dispersion and their respective size is found to be 12 nm. These results are also well matched with the FESEM images of the same nanostructures discussed earlier. The corresponding SAED patterns attained from the large area of particles are shown in Fig. 3b, d, f. Formation of rings in these patterns also reveal the existence of cubic spinel structure of γ-Fe_2_O_3_ and well correlated to the *hkl* (220), (311), (400), (422), (511) and (440) planes of X-ray diffraction study discussed later.

### Bio-surfactant assisted formation of 0D and 1D iron oxide nanostructures

The formation mechanism of 1D nanostructured γ-Fe_2_O_3_ is proposed mainly based on the chemical structure of FS (capping agent/bio-surfactant) (Supplementary Fig. S1). It seems that the growth of 1D nanostructure in a particular direction is predominantly due to two factors. The first one is the probability of adsorption of FS on the surface of these nanoparticles building blocks rendering a preferential assembly along a certain direction^28^ and secondly, the concentration of OH^−^ ions present in FS assists the growth of NGs or NWs of γ-Fe_2_O_3_. It is reported that a slow hydrolysis of the transition metal salts helps to control the branching of the building blocks and leads to a guided growth^29–31^. On the other hand, the presence of higher OH^−^ concentration enhances the agglomeration of nanocrystals to a greater extent by ascertaining the metal–oxygen–metal bonds at many different sites and locations which eventually leads to an unguided growth^32,33^.

Interestingly the inherent anisotropy of crystal structure or crystal surface reactivity is investigated in the previous reports^34,35^, and this has been identified as the driving force for the growth of 1D nanostructures. G. Michael et al. showed that well defined facets of Ag nanoparticles may have different polarizability and reactivity, which leads to the oriented formation of Ag nanowires^36^. In our work, under a certain reaction condition (2% and 4% FS), the adsorption and detachment rate of the stabilizing agent (FS) molecules on the surface of NSs is accelerated. The acceleration of these adsorption and detachment rate of FS molecules reduces the mutual electrostatic repulsion between the nanoparticles. When the attraction force between these NSs dominates over the steric repulsion, the oriented attachment of NSs occurs during the synthesis process. Thus, the product is determined by the repulsion and attractive forces between these nanoparticles. Hence it can be reasonably concluded that the competition between dipolar attraction and steric repulsion influences the growth of these nanocrystals and hence the resultant nanostructures.

On the other hand, higher concentration (10%) of the bio-surfactant strongly competes with the iron oxide nanostructures and helps to retain the nanospherical structure, where only homogeneous sphere shaped nanoparticles are formed. These bio-surfactant molecules form repulsive forces between the nucleated particles and subsequently prohibiting the further growth. The ability of these micelles to prevent the growth of these particles becomes stronger at higher surfactant concentrations and hence the average particle size decreases, finally leading to the homogeneous dispersal of the spherical nanoparticles. It also means that the distribution of bio-surfactant in various directions on the surface of these nanoparticles is highly isotropic, hence, the better size distribution of the crystal growth in the process could be obtained with more befitting amount of the surfactant used in the reaction^37^. These idiosyncratic properties of the saponin rich bio-surfactants are not only play a vital role in designing the morphology of iron oxide nanostructures and also facilitate the formation of various phases of iron oxides.

### Structural characteristics

**Figure 4:**
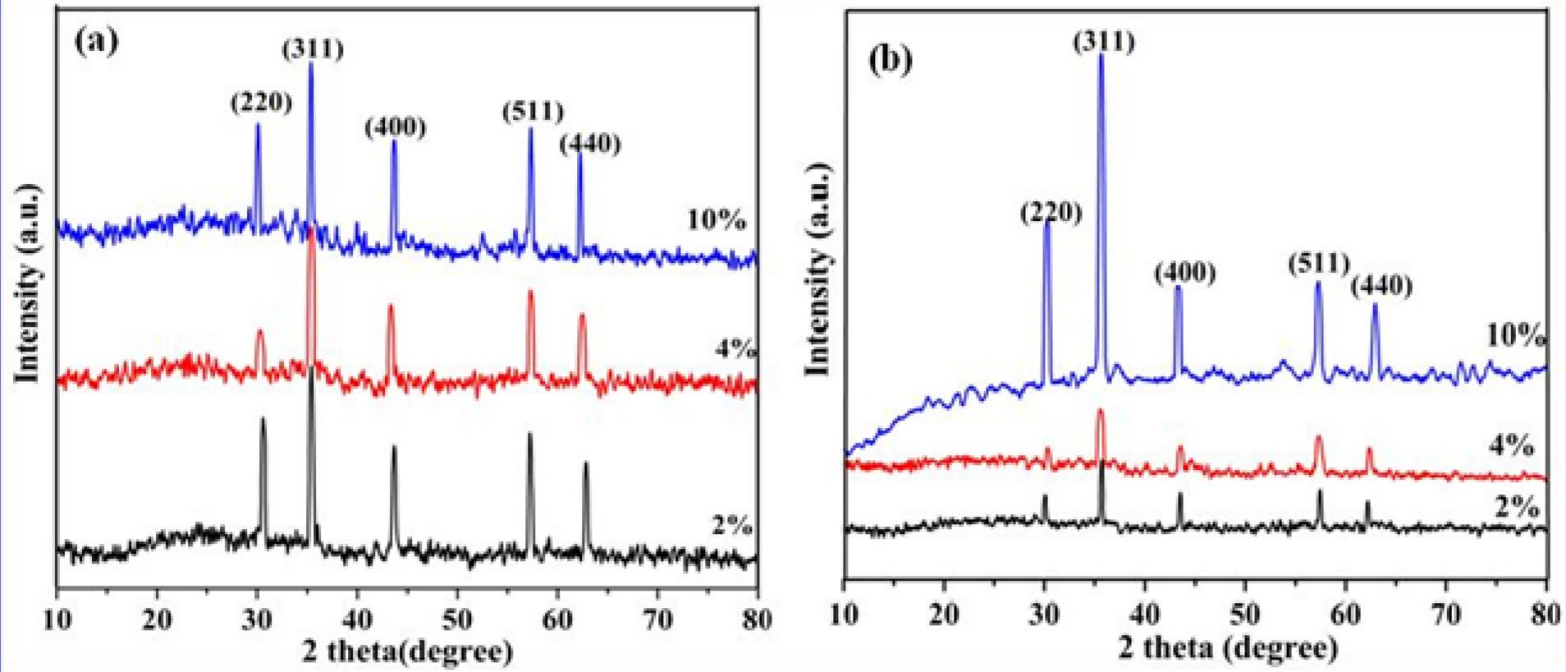
(**a**) XRD patterns of γ-Fe_2_O_3_ nanostructures synthesized at different volume % of FS using 30 min time duration. (**b**) XRD patterns of γ-Fe_2_O_3_ nanostructures synthesized at different volume % (2% , 4% and 10%) of FS for 3 h time duration.

Further to confirm the crystalline structure of resultant γ-Fe_2_O_3_ samples synthesized at different volume % (2%, 4% and 10%) of FS and for 30 min and then for 3 h time duration, XRD studies are carried out. The corresponding XRD patterns are shown in Fig. 4. Typical XRD characteristics observed for the synthesized iron oxide NSs correlate very well with the cubic inverse spinel phase of γ-Fe_2_O_3_. Figure 4a represents the XRD patterns of NSs of γ-Fe_2_O_3_ prepared for 30 min, where all the diffraction peaks appeared at 2θ values of 30.2, 35.4, 43.5, 57.3 and 62.1º corresponding to *hkl* planes of (220), (311), (400), (511) and (440) that can be indexed to the pure form of γ-Fe_2_O_3_ (JCPDS No. 89-5892) as shown in the standard XRD data (Supplementary Fig. S2). Formation of the intense peaks in XRD patterns indicates the pure crystalline nature of resultant γ-Fe_2_O_3_ products. In addition, these samples are phase pure, as the spectra did not show any traces of other impurity phase peaks of iron oxide such as α, β or δ phases or any other phases of iron oxy hydroxides, such as FeOOH, or Fe(OH)_3_ and only γ-Fe_2_O_3_ peaks are observed in XRD.

Interestingly XRD patterns of all these samples are very similar to each other, except a negligible variation in the intensity values, indicating the increased crystallinity without any phase changes. The broad nature of diffraction patterns is an indication of the formation of smaller sized particles. Figure 4b shows the XRD patterns of γ-Fe_2_O_3_ nanocrystals with three different shapes (NGs—2%, NWs—4% and NSs—10%) synthesized using different volume % of FS for 3 h, where all the spectral lines can be assigned to the pure form of cubic inverse spinel of γ-Fe_2_O_3_ (maghemite) structure (JCPDS No. 89-5892 as shown in Supplementary Fig. S2). These nanostructures of γ-Fe_2_O_3_ exhibited well-resolved diffraction peaks at 2θ values of 30.0°, 35.4°, 43.2°, and 62.7°, which are attributed to (220), (311), (400), (511), and (440) reflections corresponding to γ-Fe_2_O_3_ phase^38^.

Further XRD results showed that NGs and NWs are preferentially oriented along (311) plane. Moreover, for the increased FS content in the reaction mixture (at 10%) the structure uniformity is maintained but did not form 1D γ-Fe_2_O_3_ nanostructures as like in the samples synthesized using 2% (NGs) and 4% (NWs) FS. This can be inferred from the fact that increased number of spheroid-shaped nanoparticles is observed under FESEM and TEM analyses (Figs. 2, 3). This is also reflected from the increased height of particularly maghemite peaks in XRD studies, while the intensity values of the wires and grass shaped maghemite nanoparticles are decreased. The broad nature of these diffraction bands observed in XRD pattern recorded for 10% FS is an indication of the formation of smaller sized particles with an average size of 12 nm. It is also explained and reiterates that the use of bio-surfactant (FS) did not result in the phase change of γ-Fe_2_O_3_, but only controlled the size and shape of the resultant particles^39^. FTIR spectra of pure FS extract and different samples (NGs, NWs and NSs of γ-Fe_2_O_3_) synthesized using various volume % of FS for 30 min and 3 h time duration have been recorded to confirm the formation of Fe–O bond in these samples. FTIR spectra (Supplementary Figs. S3, S4) of all the iron oxide nanostructures consisting of the characteristic –OH stretching (ν OH) and –HOH bending (δ OH) and vibrational bands in the region between 3,100 and 3,400 cm^−1^ respectively. This could be attributed to the stretching vibrations of adsorbed moisture and the surface hydroxyl groups present on the surface of iron oxide. The sharp peaks centered at 1,100 cm^−1^ in all these spectra could be assigned to (δ C–O–H) stretching vibration of saponin molecule present in the FS extract and the peak at ~ 1,620 cm^−1^ represents pure –CH_2_ group vibration of sugar moieties found in FS. Similarly the band appeared at ~ 1,370 cm^−1^ could be attributed to the deformation vibration of C-H bond of alkane present in the FS extract. Similarly, appearance of a transmittance peak at 604 cm^−1^ corresponds to the stretching vibration of tetrahedral iron atoms (ν Fe–O).

**Figure 5:**
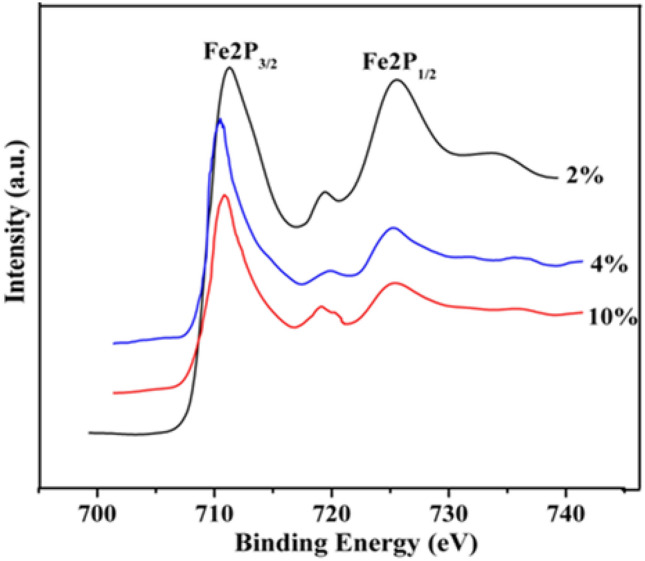
XPS spectra of γ-Fe_2_O_3_ nanostructures synthesized using different volume % (2% NGs, 4% NWs and 10% NSs) of FS for 3 h time duration.

Further XPS analysis is carried out to examine the chemical environment of the elements and the oxidation state of iron in these resultant nanostructures (NGs, NWs and NSs) since the core electron lines of both ferrous and ferric ions can be detected and are distinguishable from each other using XPS studies, which is not possible with XRD analysis. The corresponding results are shown in Fig. 5. The binding energy peaks emerged at 710.7 eV and 724.6 eV for γ-Fe_2_O_3_ NGs produced from 2% FS; 710.4 eV and 724.5 eV for the NWs prepared from 4% FS and 710.5 eV and 724.8 eV for the NSs synthesized from 10% FS concentrations are the characteristic doublet peak of Fe 2*p*_3/2_ and Fe 2*p*_1/2_ core-level electrons respectively. Formation of these core level peaks indicates the presence of + 3 oxidation state in γ-Fe_2_O_3_. Moreover, the well-resolved satellite peaks appeared at a higher binding energy side of the main doublet peaks are noted at 719.1 eV, 719.3 eV and 719.4 eV for the iron oxide samples synthesized from 2%, 4% and 10% of FS respectively. These peaks represent the fingerprints of the electronic structure of Fe^3+^ (indirectly indicates the absence of + 2 ion)^40,41^. Thus XPS results revealed that the charge transfer screening can be solely attributed to the presence of Fe^3+^ ions in γ-Fe_2_O_3_. All the above experimental results confirm that the synthesized samples are in pure form of γ-Fe_2_O_3_ rather than Fe_3_O_4_ phase^42^. Therefore, on the basis of colour, FESEM, TEM, XRD, and XPS analyses, the synthesized nanostructures are identified to be a phase pure form of cubic γ-Fe_2_O_3_ with their preferential growth oriented along (311) and (220) planes of the crystal.

### TGA/DTA analysis

**Figure 6:**
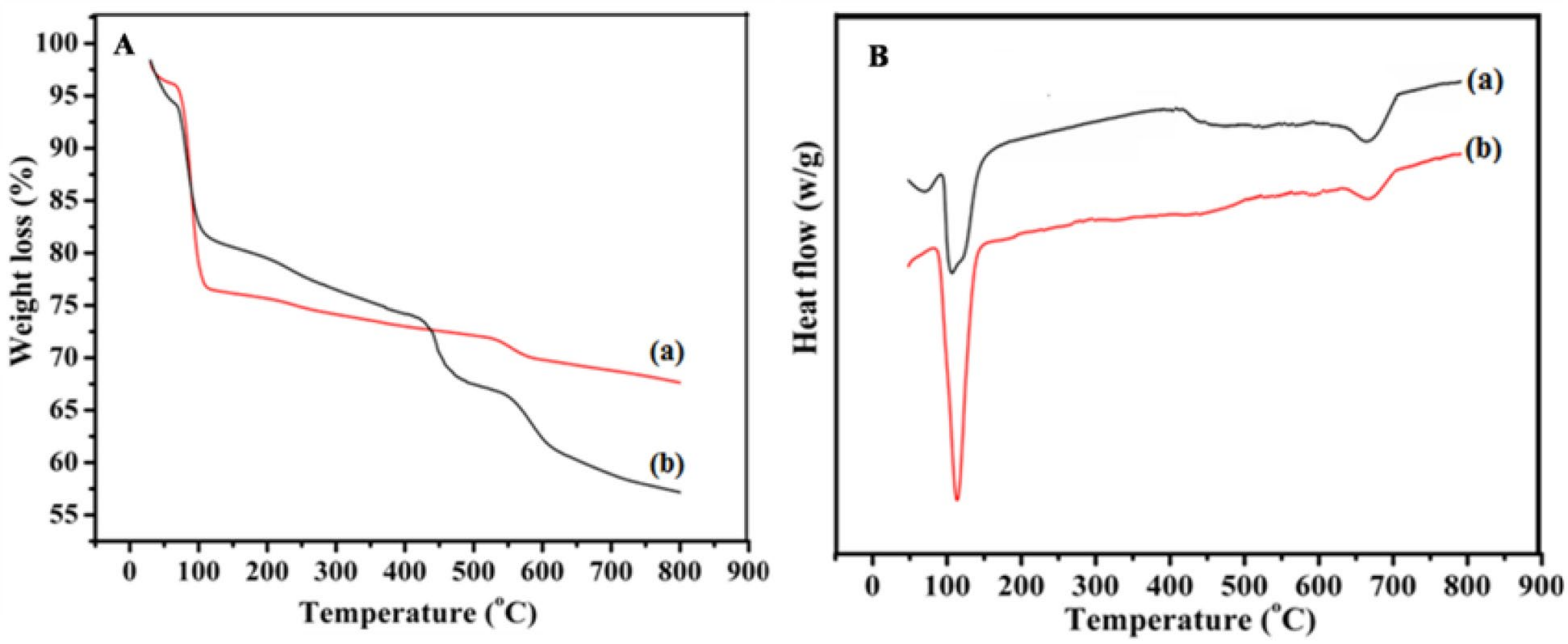
(**A**) TGA and (**B**) DTA curves of (a) γ-Fe_2_O_3_ NWs synthesized using 4% FS and (b) γ-Fe_2_O_3_ NSs synthesized using 10% FS for 3 h time duration.

Thermal gravimetric analysis (TGA) and differential thermal analysis (DTA) provide insight information about thermal and structural stability of these resultant γ-Fe_2_O_3_ nanostructures. γ-Fe_2_O_3_ NWs and NSs have been analyzed for the structural stability through TGA/DTA analysis from 30 to 800 °C under N_2_ atmosphere and their corresponding curves are displayed in Fig. 6A, B. TGA curve undergoes a three stage weight losses and the first one occurs below 110 °C due to the removal of adsorbed water (surface –OH groups) which is more (20%) for γ-Fe_2_O_3_ NSs (b) compared to γ-Fe_2_O_3_ NWs (a), where only 15% weight loss is noted. However this higher percentage weight loss observed for NSs suggested the conversion of oxyhydroxide forms to oxide of iron. The second comparatively smaller weight loss of 1.93% can be attributed to the structural arrangement of iron oxide. Consequently, 2nd and 3rd stage weight loss can be attributed to the decomposition of organic moieties present in the FS and phase transformation of γ-Fe_2_O_3_, where higher weight loss for the NSs can be seen because of the more amounts of organic moieties present in the extract (10% FS) adsorbed on the surface of these NSs.

Figure 6B represents the broad endothermic peaks at around 120 °C observed in both the DTA curves and is mainly ascribed to the removal of physically bounded water molecules. The above mentioned characteristics arises from the finite size effect, where the surface availability on the nanostructures for the anchoring of water molecules (–OH) play a vital role. On increasing the temperature, the noticeable endothermic peak observed between 600 and 700 °C in the DTA curve is also associated with the transformation of nanosized γ-Fe_2_O_3_ phase. This implies that the thermal stability of the synthesized γ-Fe_2_O_3_ nanostructure is very high; usually the transformation occurs between 450 and 550 °C for the bulk samples. However, the disappearance of obvious exothermic peak at around 640 °C in the DTA curve of both these samples, representing no phase transition occurs from γ-Fe_2_O_3_ to α-Fe_2_O_3_^43^. Thus, the present bio-surfactant assisted co-precipitation method elucidated its uniqueness by tailoring the stable phase of γ-Fe_2_O_3_ nanostructures under simple experimental conditions.

### Investigation of magnetic properties

**Figure 7:**
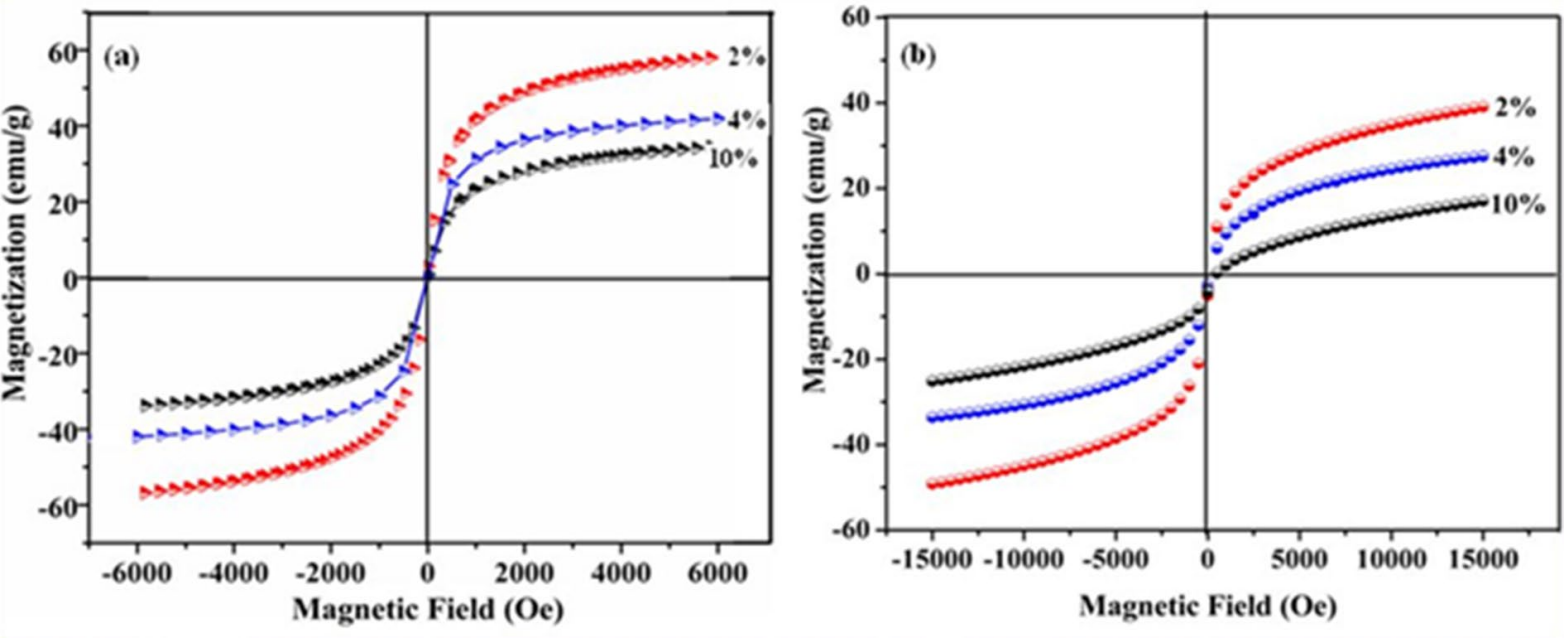
(**a**) M-H curves of γ-Fe_2_O_3_ nanostructures synthesized at different volume % (2, 4 and 10%) of FS for 30 min time duration. (**b**) M-H curves of γ-Fe_2_O_3_ nanostructures synthesized using different volume % (2% NGs, 4% NWs and 10% NSs) of FS for 3 h time duration.

Furthermore, the effects of size and shape modulation of the resultant nanostructured materials of γ-Fe_2_O_3_ on their magnetic properties are investigated for the synthesized samples and their corresponding hysteresis loops are depicted in Fig. 7. The room-temperature hysteresis loops for a series of samples synthesized from various volume % (2%, 4% and 10%) of FS extract for 30 min (Fig. 7a) and for 3 h (Fig. 7b) time duration have been recorded. These curves exhibit neither remanence nor coercivity, regardless of the nanocrystal size and shape and therefore indicate a superparamagnetic behaviour. The saturation magnetization (M_s_) values of all these synthesized γ-Fe_2_O_3_ samples fall below that of bulk γ-Fe_2_O_3_ value of 74 emu/g, which provide further evidence for the phase purity of these materials^44^. The room temperature M_s_ values of different nanostructured materials synthesized at 2%, 4% and 10% of FS using 30 min are measured to be 57, 41 and 34 emu/g respectively. On the other hand, the measured M_s_ values for γ-Fe_2_O_3_ NGs, γ-Fe_2_O_3_ NWs synthesized using 2% and 4% of FS for 3 h time duration are determined to be 39 and 28 emu/g respectively. The slope of the magnetization is positive even at larger applied fields with an unending hysteresis. This is attributed mainly to the formation of 1D nanostructures that are finite, non-ellipsoidal particles and cannot be saturated by a homogeneous applied field. For γ-Fe_2_O_3_ NSs produced from 10% FS, the M_s_ value is further reduced to 16 emu/g as the size decreased nearing to that of quantum dots^45–47^.

These results indicate the single domain magnetic characteristics of the nanoparticles that remained same for the NSs synthesized using 30 min and 3 h (10% FS) time duration. Since the amount of all these samples used for the measurement of magnetic properties was kept constant, the decrease of saturation magnetization is mainly ascribed to the increased amount of bio-surfactant (FS) covered over the maghemite nanoparticles that hinders the magnetic susceptibility. Hence, the notable decrease in the saturation magnetization is most likely due to the existence of FS on the surface of γ-Fe_2_O_3_ nanoparticles which may create a magnetically dead layer. With a significant fraction of surface atoms on these nanoparticles and any crystalline disorder within the surface layer may also lead to a significant decrease in the saturation magnetization values of these nanoparticles^46^. Thus the magnetic characteristics of these synthesized iron oxide nanostructures could be influenced by the shape, size and concentration of the bio-surfactant used for the preparation.

### Electrochemical sensing of DA and UA using nanostructures of γ-Fe_2_O_3_

**Figure 8:**
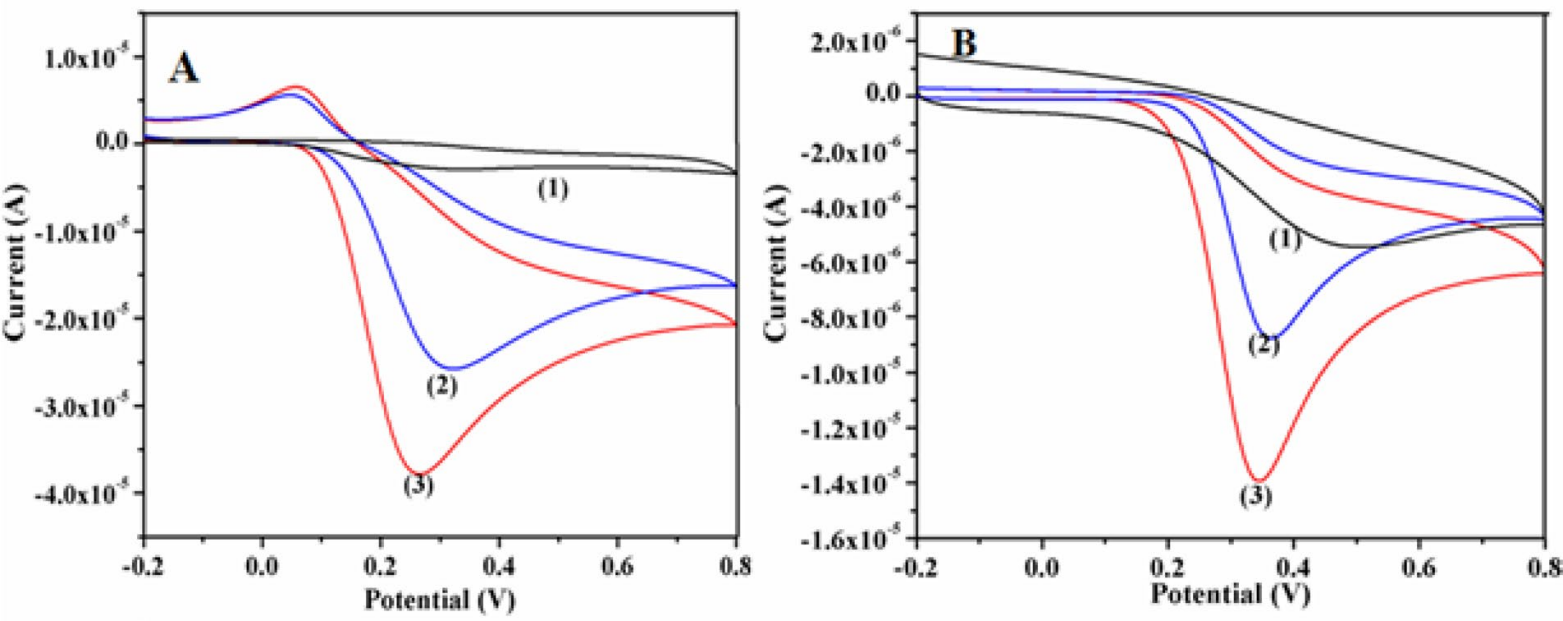
Cyclic voltammetric responses obtained using (1) bare GCE, (2) γ-Fe_2_O_3_ NSs and (3) γ-Fe_2_O_3_ NWs modified GCEs in PBS (pH = 7.4) at a fixed scan rate of 50 mV s^−1^ for (**A**) 0.5 mM DA and (**B**) 0.5 mM UA respectively.

Different nanostructures of γ-Fe_2_O_3_ viz., NGs, NWs and NSs are explored further for the electrochemical sensing application for the simultaneous detection of DA and UA. The effects of shape and size of these nanostructures on the electrochemical sensing characteristics are also investigated. Electrocatalytic activity of these γ-Fe_2_O_3_ nanostructures was studied by coating these materials onto pre-cleaned glassy carbon electrode (GCE) surface and their electrochemical oxidation capability towards DA and UA was analyzed. These studies are carried out using γ-Fe_2_O_3_/GCE through cyclic voltammetry (CV) technique within the potential range of − 0.2 V to + 0.8 V in aqueous phosphate buffer solution (PBS) having pH = 7.4. Among the different nanostructures studied in this work, NWs and NSs of γ-Fe_2_O_3_ found to exhibit better electrochemical sensing capability for DA and UA (Supplementary Fig. S5). Hence the subsequent studies were carried out with these nanostructures. The corresponding cyclic voltammograms recorded for 0.5 mM DA and 0.5 mM UA using NWs and NSs of γ-Fe_2_O_3_ are shown in Fig. 8A, B respectively and for comparison similar studies performed using bare GCE were also shown in the figure. The individual electrochemical oxidation of both DA (Fig. 8A) and UA (Fig. 8B) on γ-Fe_2_O_3_ nanostructures modified GCE exhibited negative shift of anodic potentials with increased current response when compared to bare GCE. The electrocatalytic oxidation of DA is evident from the oxidation peak appeared at 290 mV, 250 mV and 300 mV for NSs and NWs of γ-Fe_2_O_3_ modified GCEs along with a bare GCE, respectively. For the electrochemical detection of UA, the oxidation peaks appeared at 350 mV and 330 mV for NSs and NWs of γ-Fe_2_O_3_ modified GCEs, whereas in the case of bare GCE the voltammetric response for UA appeared at a higher potential of 520 mV and no obvious cathodic peak potential are observed in all these cases, suggesting the irreversibility of the electrochemical process^36,48,49^. Various electrochemical characteristic parameters are determined for these electrodes from their corresponding CVs (Supplementary Table S1).

Interestingly the current response observed for the electrocatalytic activity of DA and UA at γ-Fe_2_O_3_ NSs modified GCE is slightly lower than that of γ-Fe_2_O_3_ NWs modified GCE. On the other hand, oxidation peak current values noted at γ-Fe_2_O_3_ NSs and NWs modified GCEs are greater than that of bare GCE. These results indicate the fast electron transfer kinetics of DA and UA on γ-Fe_2_O_3_ nanostructures coated GCEs. The major reason is that the synthesized γ-Fe_2_O_3_ NWs can act as a catalyst to increase the rate of electron transfer and lower the overpotential for oxidation of DA and UA. Moreover the fast response resulted from the excess electro-active sites provided by γ-Fe_2_O_3_ NWs as well as the good conductivity between the coated film and GCE substrate. The reason for this improved electrocatalytic activity is mainly attributed to the 1D structure of γ-Fe_2_O_3_ NW, which acts as an “electron wire”, wherein the electron diffusion occurs at a faster rate that aids in increased sensitivity towards the sensing of DA and UA. Such a nanostructure is more favourable for providing a large contact area between the sensing materials and the analyte species than non-hierarchical nanoparticles or bulk Fe_2_O_3_ powders. Furthermore, due to the small size of these NWs, the charge distribution on the surface may lead to less resistance to the diffusion of probe ions onto the electrode surface than that of the bulk Fe_2_O_3_.

Further the material exhibits a facile redox process associated with Fe within this potential window that might result in enhanced conductivity both in terms of ionic and electronic transport phenomena. Such kind of redox process involving Fe(III)/Fe(II) centers of γ-Fe_2_O_3_ accelerates the electrocatalytic mechanism behind the sensing of these bio-analytes. Consequently, γ-Fe_2_O_3_ NWs modified GCE projected as a sensing platform for the oxidation of DA and UA exhibits more electro-active sites and strong adhesion onto the surface, resulting in the enhanced sensitivity and shows a lower overpotential for the sensor. Finally, from these results it is clear that γ-Fe_2_O_3_ NWs aid in more effective transportation and accessibility of bio-analytes and subsequently facilitates the electron transfer process that ultimately leads to less non-faradaic behaviour with pronounced sensitivity^50^.

### Optimization of pH

**Figure 9:**
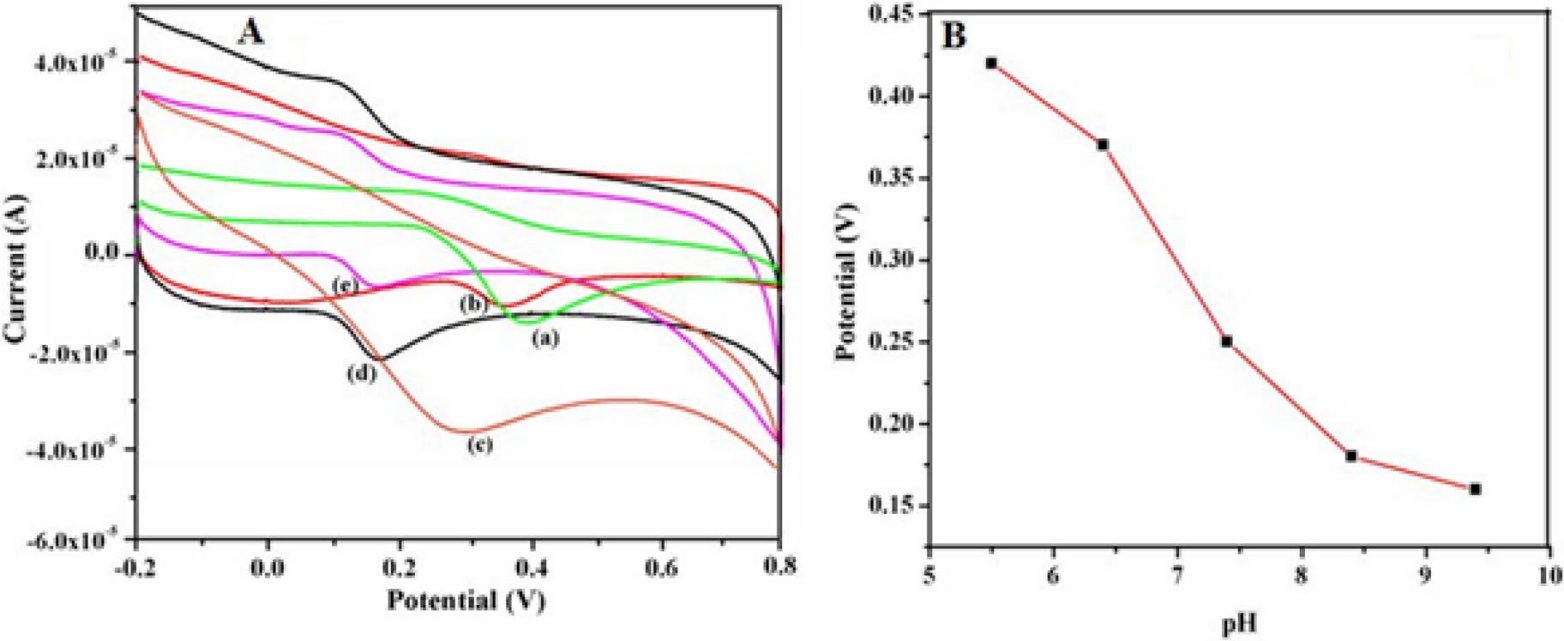
(**A**) Cyclic voltammograms recorded at a fixed scan rate of 50 mV s^−1^ using 0.5 mM DA at different pH values namely, (a) 5.4, (b) 6.4, (c) 7.4, (d) 8.4 and (e) 9.4 respectively at γ-Fe_2_O_3_ NWs modified GCEs. (**B**) Change in DA oxidation potential with respect to pH of the solution.

**Figure 10:**
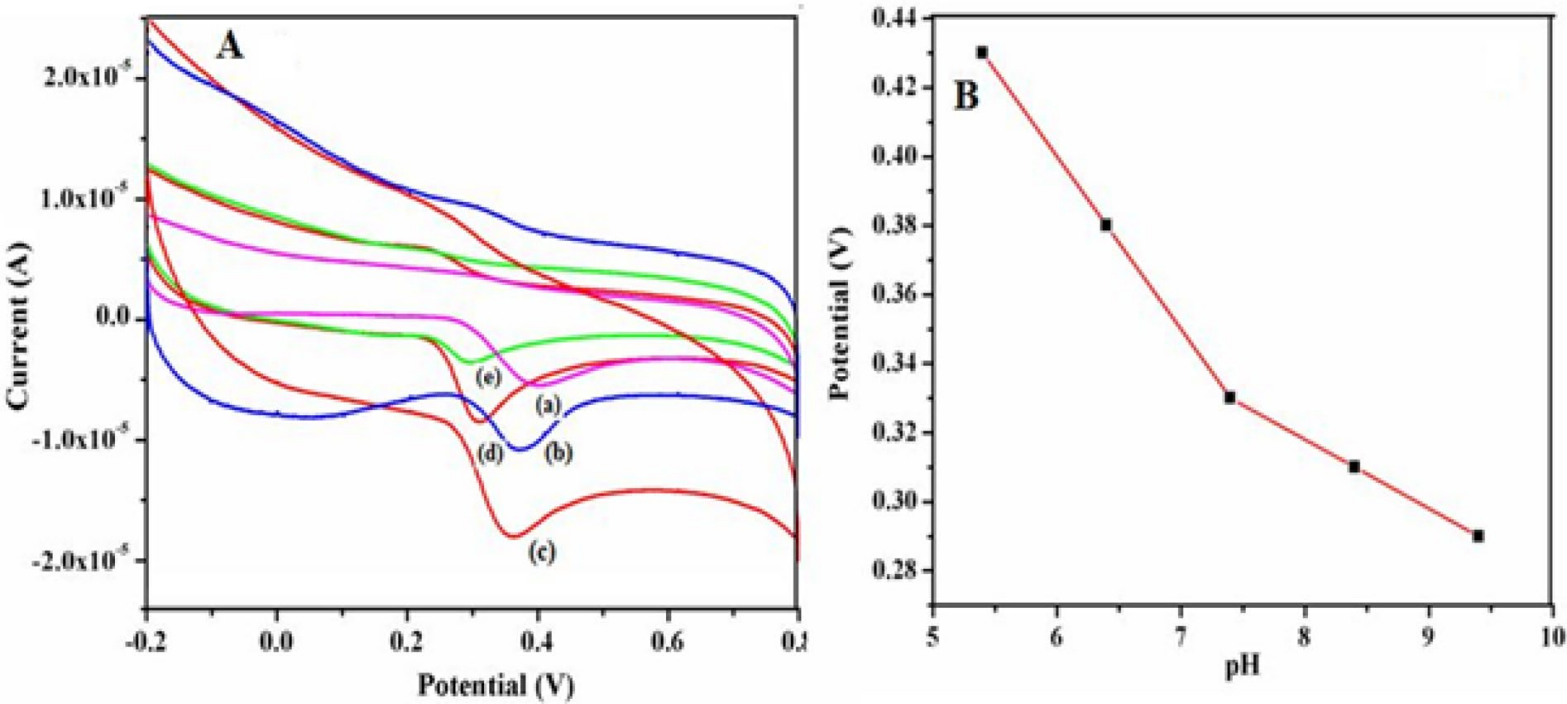
(**A**) Cyclic voltammograms recorded at a fixed scan rate of 50 mV s^−1^ using 0.5 mM UA at different pH values namely, (a) 5.4, (b) 6.4, (c) 7.4, (d) 8.4 and (e) 9.4 respectively at γ-Fe_2_O_3_ NWs modified GCEs. (**B**) Change in UA oxidation potential with respect to pH of the solution.

It is found that pH values affect the electrochemical signals of DA and UA at γ-Fe_2_O_3_ NWs modified GCEs and hence the optimization studies were carried out over different pH values ranging from 5.4 to 9.4 and the corresponding results are shown in Figs. 9 and 10. These studies are performed to determine the optimum pH value at which the highest electrochemical responses for the oxidation of DA and UA are achieved. From these results it is found that the oxidation peak current of DA reaches the maximum value at pH 7.4 and then drops quickly and finally decreases with increasing pH. Similarly, the peak current of UA also initially goes up gradually with increasing pH (5.4–7.4), and then it decreases with increasing pH (> 7.4). In addition, Figs. 9B and 10B show the variation of peak potentials of both DA and UA oxidation with pH and these plots exhibit a pH dependency. The oxidation peak potential shifted to less negative value when the pH of the solution is increased, which indicates that the electrocatalytic oxidation of DA and UA at these modified electrodes involves equal number of electrons and protons^51,52^. Meanwhile, increasing pH also leads to a sharp decrease in the oxidation current when pH is more than 7.4 (Figs. 9A, 10A). Moreover, the maximum oxidation current response is observed at pH of 7.4 for the determination of DA and UA at γ-Fe_2_O_3_ NWs modified electrodes and hence it is adopted as the optimum pH for further experiments.

### Effect of scan rate

**Figure 11:**
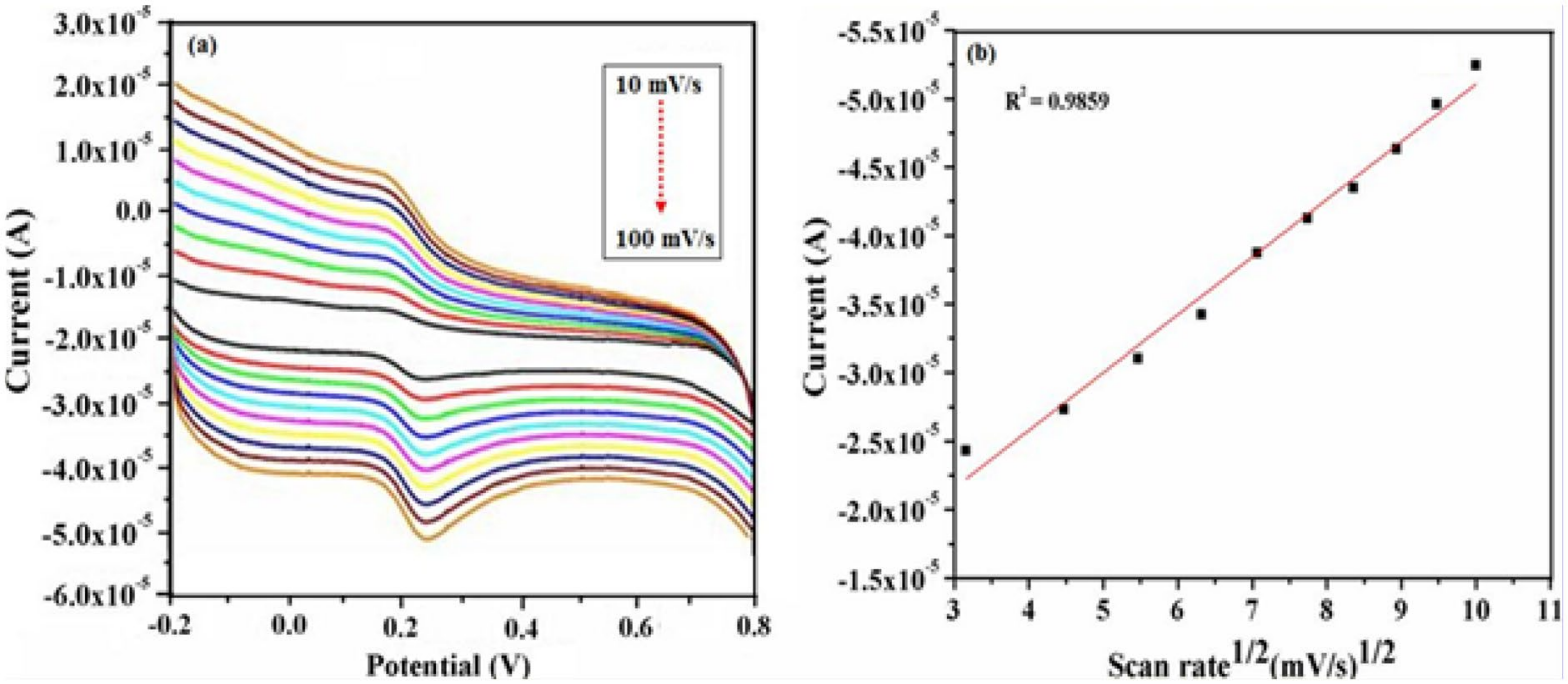
(**a**) Cyclic voltammograms recorded using 0.5 mM DA in PBS (pH—7.4) solution over different scan rate values ranging from 10 mV/s to 100 mV/s at γ-Fe_2_O_3_ NWs modified GCEs. (**b**) Calibration curve of the anodic oxidation peak current response with respect to square root of scan rate. In this figure the arrow indicates the direction of increasing scan rate.

**Figure 12:**
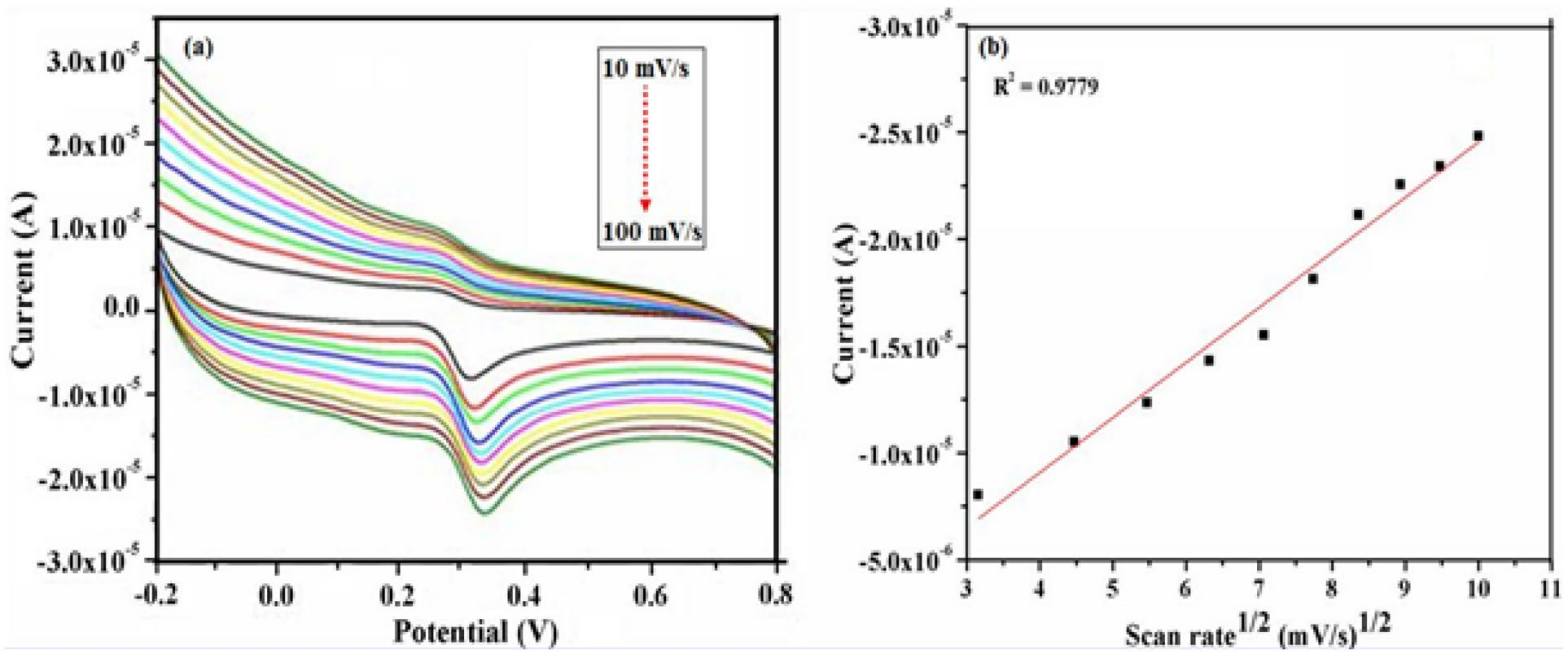
(**a**) Cyclic voltammograms recorded using 0.5 mM UA in PBS (pH—7.4) solution over different scan rate values ranging from 10 mV/s to 100 mV/s at γ-Fe_2_O_3_ NWs modified GCEs. (**b**) Calibration curve of the anodic oxidation peak current response with respect to square root of scan rate. In this figure the arrow indicates the direction of increasing scan rate.

The influence of scan rate on the oxidation potential and subsequently the kinetics of electron transfer associated with oxidation of DA and UA is investigated using γ-Fe_2_O_3_ NWs modified GCEs. These studies are carried out for a wide range of scan rate varying from 10 mV/s to 100 mV/s using CV within the potential range of − 0.2 V to + 0.8 V. Figures 11 and 12 show the respective CV curves of 0.5 mM DA and 0.5 mM UA recorded using γ-Fe_2_O_3_ NWs modified GCEs at different scan rates. It can be seen that the anodic peak current values of DA are proportional to the square root of scan rate in the range from 10 to 100 mV/s with a correlation coefficient of R^2^ = 0.9859 (Fig. 11b). Meanwhile, the anodic peak current values of UA also showed an excellent linear relationship with the square root of scan rate in the range from 10 to 100 mV/s with a correlation coefficient of R^2^ = 0.9779 (Fig. 12b). As shown in these figures, with increasing scan rate, the redox current values increased and good linear relationships are established between the peak current values and the square root of scan rate, respectively, indicating that the oxidation of DA and UA on γ-Fe_2_O_3_ NWs modified GCE is a diffusion controlled process^53^.

### Electrochemical sensing of DA using DPV studies

**Figure 13:**
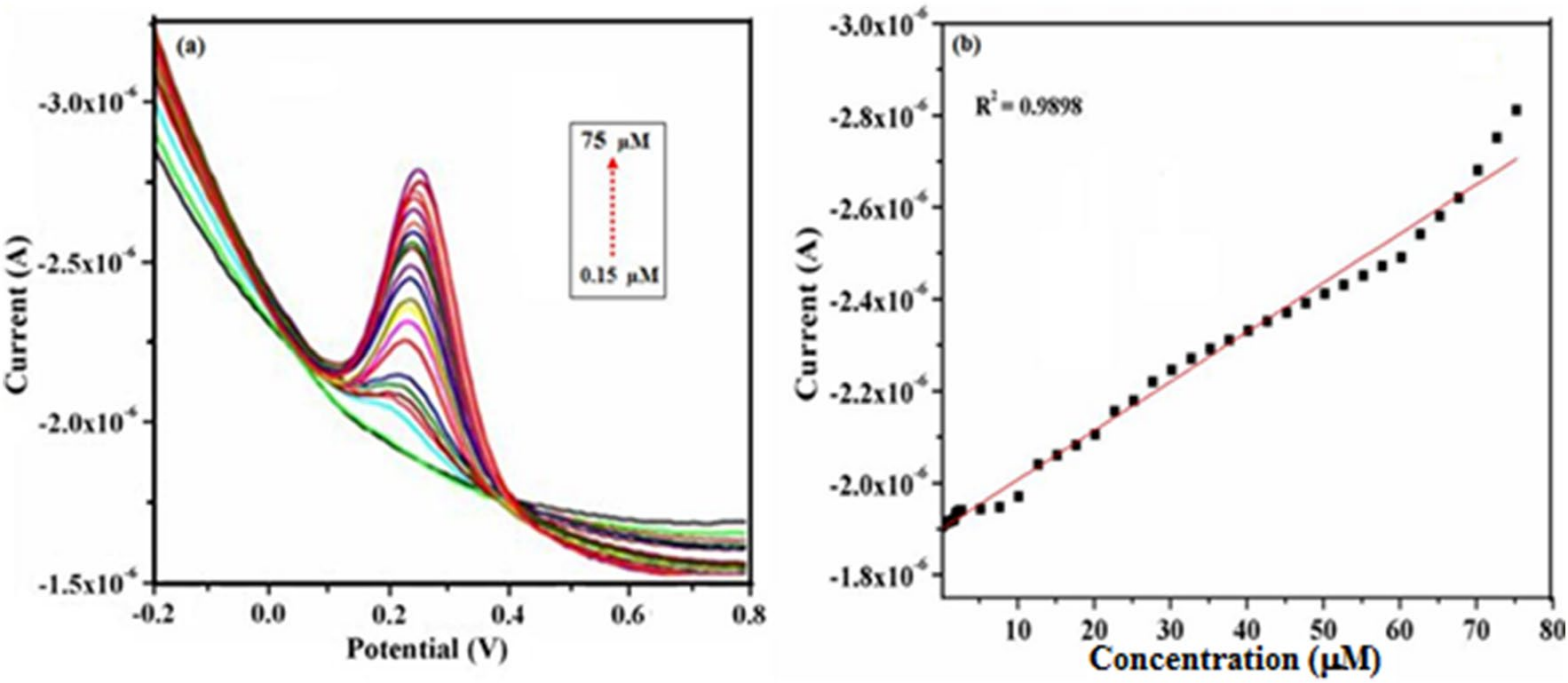
(**a**) DPV responses obtained for various concentrations of DA (0.15–75 µM) in PBS (pH—7.4) solution at a fixed scan rate of 50 mV s^−1^ using γ-Fe_2_O_3_ NWs modified GCE and (**b**) Calibration curve of the anodic peak current response with respect to the concentrations of DA.

Differential pulse voltammetry (DPV) technique provides a higher sensitive current and better detection limit when compared to CV and hence the determination of DA was carried out by using DPV. Figure 13a depicts the DPV responses of γ-Fe_2_O_3_ NWs modified GCE for the addition of different concentrations of DA from 0.15 μM to 75 μM. It can be seen that a sharp DPV response is observed for the addition of 0.15 μM DA and the current response increased linearly with increasing concentrations of DA. Interestingly DPV response of γ-Fe_2_O_3_ NWs/GCE is linear over the concentration of DA ranging from 0.15 μM to 75 μM (Fig. 13b) with the correlation coefficient of R^2^ = 0.9898. This study displayed a highest linear range of detection for DA and the detection limit is determined to be 0.1 μM. The sensor characteristic parameters of the fabricated system have also been compared with the other reported DA sensors and the comparative results are shown in Table 1. The analytical comparison of our proposed sensor clearly reveals that γ-Fe_2_O_3_ NWs modified GCE exhibits a lower detection limit and better linear concentration range for the detection of DA on comparison to many other modified electrodes^54–64^.

**Table 1:** Comparison of sensor characteristics of dopamine sensor proposed in this work based on γ-Fe_2_O_3_ NWs with that of other electrode materials reported in literature.

Electrode material	Potential (V)	Detection limit (µM)	Linear range (µM)	Electrolyte (pH)	References
3D-GN@WO_3_ nanowires	0.30	238	10,000–15,000	7.2	^54^
Graphene/EPPG	0.29	3.78	5.0–50	7.0	^55^
PA6/PAH_MWCNTs nanofibers	0.19	0.15	1.0–7.0	7.0	^56^
Au–graphene	0.28	1,860	5,000–10,000	6.0	^57^
CS − GS/GCE	0.16	0.14	1.0–700	7.4	^58^
S-Fe_2_O_3_ − GCE	0.16	0.20	0.2–1.07	7.0	^59^
3DGH-Au NPs/GCE	0.29	1.0	1.0–60	7.0	^60^
Ag − Pt/pCNFs/ GCE	0.20	0.11	10–500	7.0	^61^
N-doped graphene	0.28	0.25	0.5–170	6.0	^62^
Au-AGR-MWCNT/GCE	0.29	1.4	2.0–120	7.0	^63^
2D hexagonal boron nitride/GCE	0.26	0.65	3.0–75	7.4	^64^
γ-Fe_2_O_3_ nanowires/GCE	0.25	0.15	0.15–75	7.4	This work

**Scheme 1:**
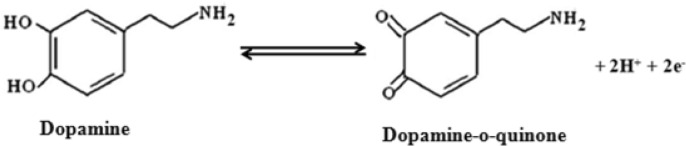
A possible mechanism proposed for the oxidation of DA using γ-Fe_2_O_3_ NWs modified GCE.

This electrocatalytic effect of DA is mainly attributed to the larger available surface area of the modifying layer due to the nanometer size of the sample and free surface –OH groups of γ-Fe_2_O_3_ as seen in FTIR studies, which could form hydrogen bonding with the –OH group of DA. It essentially contributes to the weakening of hydroxyl bond to facilitate the electron transfer through (hydroxyl group of DA) O—H–O (surface hydroxyl groups of γ-Fe_2_O_3_). Additionally, the induced magnetization of γ-Fe_2_O_3_ nanoparticles domains on applying potential results in increased magnitude of current response when compared to that of bare GCE^65^. On the other hand, γ-Fe_2_O_3_ NWs can act as an electron transfer mediator and enhances electron transfer during electrochemical oxidation of DA, as a result it becomes easier. The introduction of γ-Fe_2_O_3_ NWs onto the GCE surface facilitates the conduction pathway at the modified electrode surface. It is also reported that the redox process of DA proceeds via 2e^−^, 2H^+^ process^66^. Based on the above discussion and the previous reports, the electrochemical redox mechanism of DA at γ-Fe_2_O_3_ NWs modified GCE can be explained as shown in Scheme 1.

### Electrochemical sensing of UA using DPV studies

**Figure 14:**
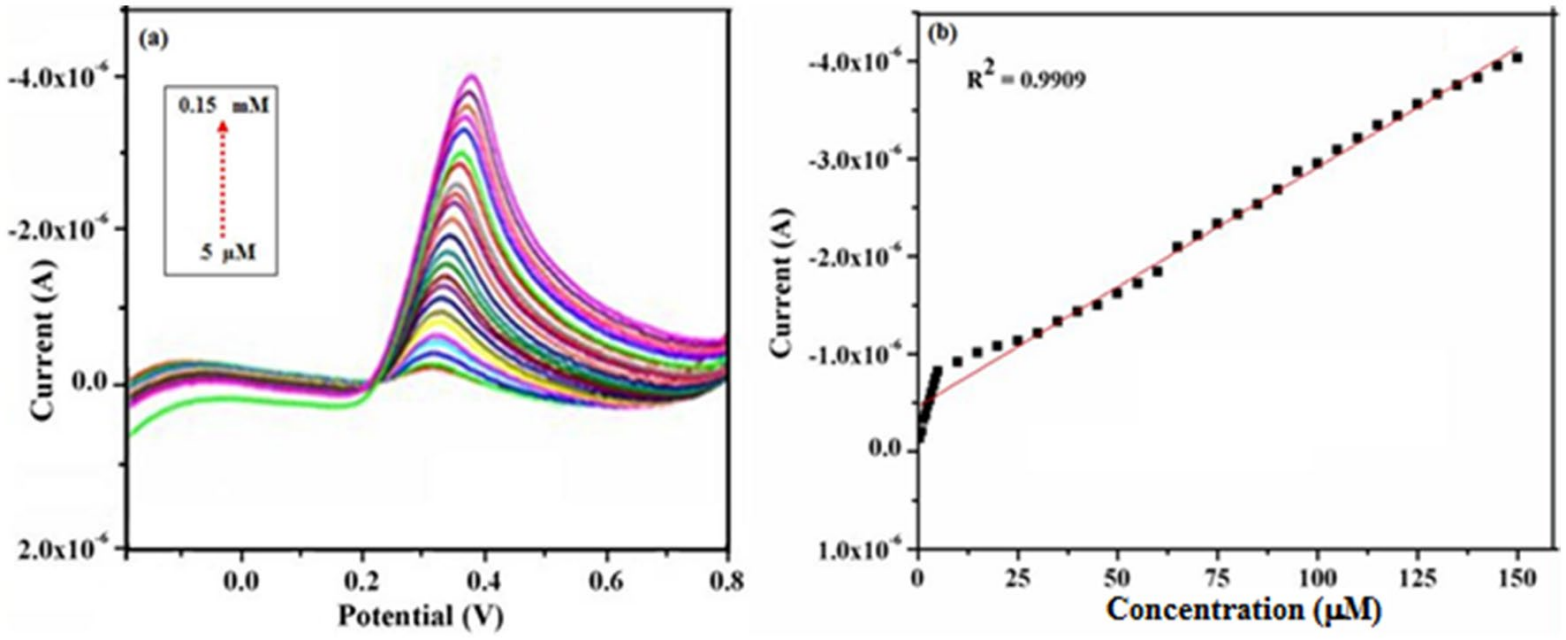
(**a**) DPV responses obtained for various concentrations of UA (5 μM–0.15 mM) in PBS (pH—7.4) solution at a fixed scan rate of 50 mV s^−1^ using γ-Fe_2_O_3_ NWs modified GCE and (**b**) Calibration curve of the anodic peak current response with respect to the concentrations of UA.

Further DPV curves were also recorded for various concentrations of UA using γ-Fe_2_O_3_ NWs modified GCE and subsequently to determine the calibration curve for UA sensor. The corresponding DPV responses are shown in Fig. 14a for the addition of various concentrations of UA ranging from 5 μM to 0.15 mM. Figure 14b represents the calibration curve for the determination of UA at γ-Fe_2_O_3_ NWs modified GCE. From these figures it is found that the response for UA oxidation increases with the increasing concentration of UA. Linear dependency could be witnessed between the oxidation current response and the concentration of UA in the range of 5 µM–0.15 mM with a correlation coefficient of R^2^ = 0.9909, suggesting a potential application of γ-Fe_2_O_3_ NWs modified GCE in the quantitative determination of UA. In comparison with the other values reported in literature, this enzyme-free electrode exhibits a wider linear concentration range than the other modified electrodes (Table 2). Furthermore, a lower detection limit of 0.5 μM UA is determined at a signal-to-noise ratio of 3 and also stipulates the minimum detectable amount of analyte using the developed sensor^67–77^.

**Table 2:** Comparison of sensor parameters of uric acid sensor proposed in this work based on γ-Fe_2_O_3_ NWs with that of other electrode materials reported in literature.

Electrode material	Potential (V)	Detection limit (µM)	Linear range (µM)	Electrolyte (pH)	References
CoTe/GP electrode	0.43	0.895	10–120	6.0	^67^
MoS_2_/PEDOT/GCE	0.39	0.95	2–25	7.4	^68^
AuNPs@MoS_2_/GCE	0.29	10.0	10–7,000	7.0	^69^
Graphene flowers/CFE	0.36	3.98	3.98–371.4	7.0	^70^
GOU/GCE	0.47	3.45	20–154	6.5	^71^
Nafion/Uricase/ZnO NSs/Ag/Si electrode	0.40	50.0	50–500	7.0	^72^
Pd@Fe_3_O_4_ electrode	0.42	0.41	0.96–107	6.8	^73^
PAYR/CPE electrode	0.40	9.50	27.8–304.4	7.0	^74^
Au–Ag NPs/GO/TH@GCE	0.45	0.30	1.0–100	7.0	^75^
GONRs + PEDOT:PSS electrode	0.33	0.50	0.5–1,200	7.0	^76^
Graphene/SnO_2_/GCE	0.40	3.0	3–21	6.8	^77^
γ-Fe_2_O_3_ nanowires/GCE	0.33	0.50	5–150	7.4	This work

**Scheme 2:**
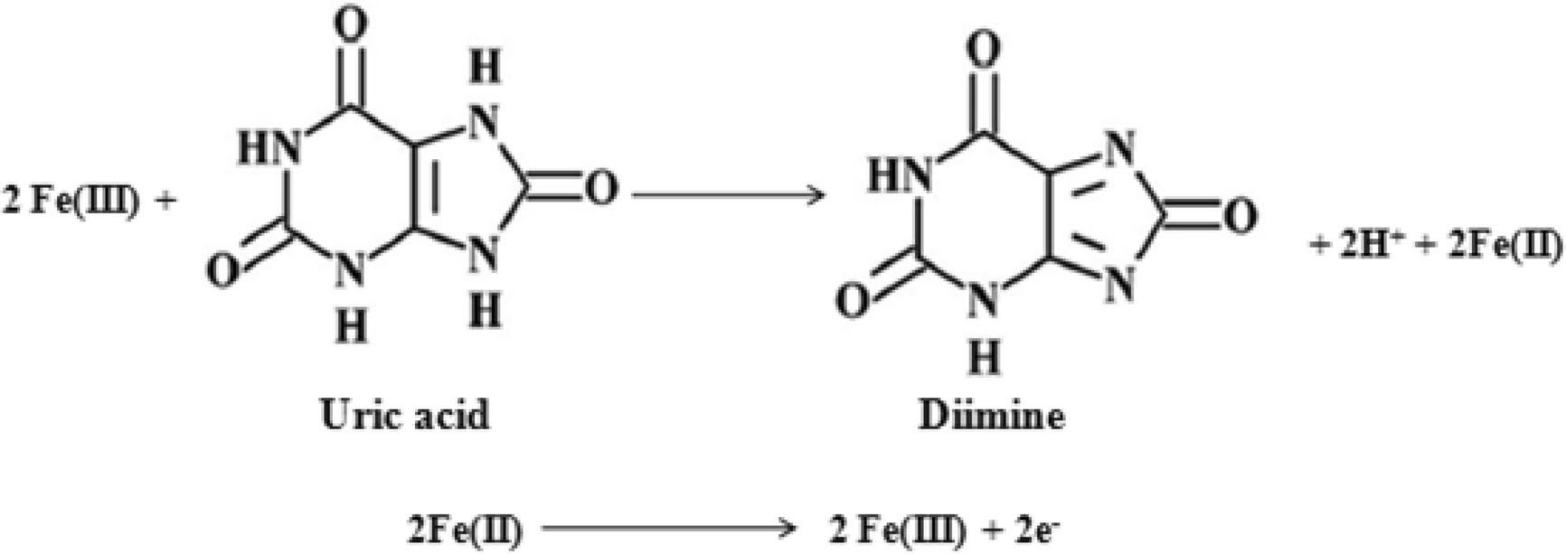
A possible mechanism proposed for the oxidation of UA on γ-Fe_2_O_3_ NWs modified GCE.

Thus the experimental results suggested that γ-Fe_2_O_3_ NWs can enhance the electron transfer rate and lower the overpotential of UA oxidation. This enhanced electrochemical sensing property is mainly attributed to the larger electro-active sites of the modifying layer due to the large number of NWs on the sample as evident from FESEM and TEM analyses. Furthermore, γ-Fe_2_O_3_ layer contains free surface –OH groups that could form hydrogen bonding with UA. It is well known that hydrogen bond acceptor strength of amide group is stronger than the ester group and it facilitates easier oxidation of UA at γ-Fe_2_O_3_ NWs modified electrode surface. Hence, the carbonyl group at C-8 of UA forms a hydrogen bonding with surface –OH groups of γ-Fe_2_O_3_ NWs. It is also evident that only Fe^2+^/Fe^3+^ redox couple is electro-active and hence Fe site plays a vital role in the electrocatalytic oxidation of UA. Recall that the oxidation of UA is irreversible at these electrodes and the oxidation of UA proceeds via 2e^−^, 2H^+^ process^68^. According to the previous reports, the electrochemical oxidation mechanism of UA at γ-Fe_2_O_3_ NWs/GCE can be explained as depicted in Scheme 2.

As shown in Tables 1 and 2, γ-Fe_2_O_3_ NWs modified sensor exhibits a better detection limit and a wider linear concentration range of detection in comparison to most of the other reported sensors. Even though the detection limit of our modified electrode is not as low as few other reported sensors, this particular one displayed acceptable results with a positive gain in the potential shift and sufficient enough to detect the physiologically relevant concentrations of the target analytes. Moreover, the synthesis protocol reported here for the preparation of iron oxide nanostructures is facile, environmentally benign method when compared to others, which are mainly based on hazardous chemical reactions.

### Simultaneous electrochemical determination of DA and UA using nanostructures of γ-Fe_2_O_3_

**Figure 15:**
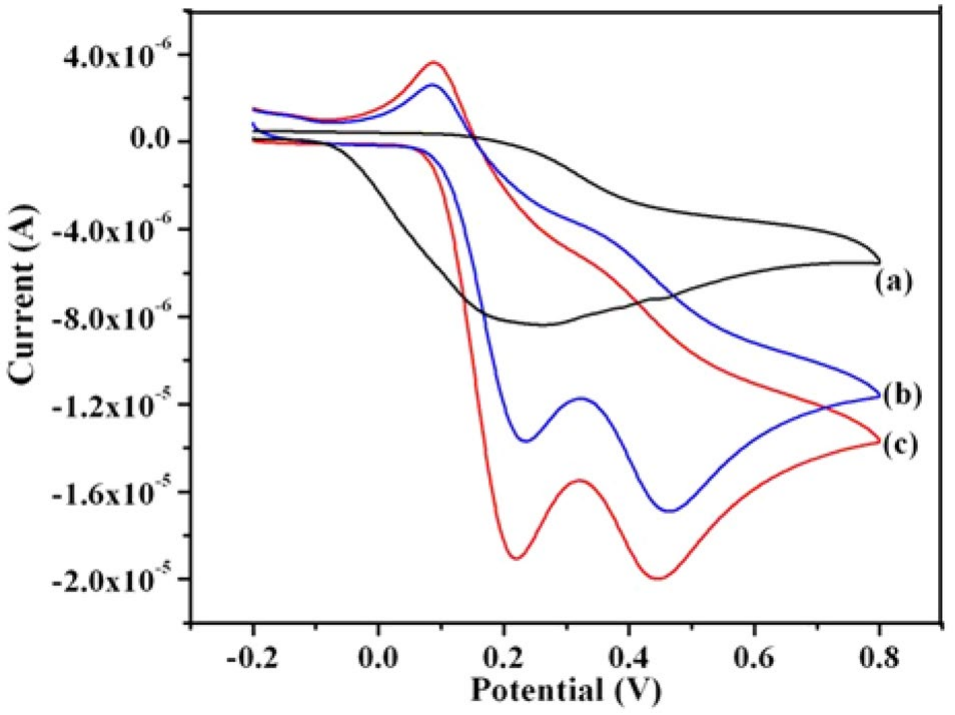
Cyclic voltammograms corresponding to the simultaneous determination of DA and UA using (**a**) bare GCE, (**b**) γ-Fe_2_O_3_ NSs and (**c**) γ-Fe_2_O_3_ NWs modified GCEs. Electrolyte: 0.5 mM DA and 0.5 mM UA in PBS (pH = 7.4) aqueous solution at a fixed sweep rate of 50 mV s^−1^.

Furthermore to demonstrate the potential utility of these γ-Fe_2_O_3_ nanostructures coated GCEs in sensing application, the simultaneous electrochemical detection of DA and UA is performed in PBS (pH—7.4) solution. These studies are carried out in a mixture of 0.5 mM DA and 0.5 mM UA and their respective cyclic voltammograms are shown in Fig. 15. It can be seen from these CVs that for a binary mixture of DA and UA, a well-defined, two separate anodic peaks corresponding to oxidation of DA and UA are observed at γ-Fe_2_O_3_ nanostructures modified GCEs. The presence of functional groups on iron oxide (γ-Fe_2_O_3_) nanostructures are able to resolve the mixed voltammetric response of these species (DA and UA) into two well resolved voltammetric peaks at the potential of 220 mV and 450 mV on γ-Fe_2_O_3_ NSs along with 200 mV and 430 mV on γ-Fe_2_O_3_ NWs modified GCEs respectively. These CV results are analyzed, and various electrochemical sensor parameters are determined (Supplementary Table S2). Further the anodic peak potential separation between DA and UA is estimated to be 230 mV and is sufficient enough to identify them as a well-defined two separate peaks. Among different samples, γ-Fe_2_O_3_ NWs showed better performance in terms of better peak separation and enhanced current values. Thus the simultaneous oxidation of DA and UA shows that γ-Fe_2_O_3_ nanostructures coated GCEs exhibit excellent electrocatalytic behavior of these species.

### Selectivity studies for the detection of DA and UA using γ-Fe_2_O_3_ NWs modified GCE

**Figure 16:**
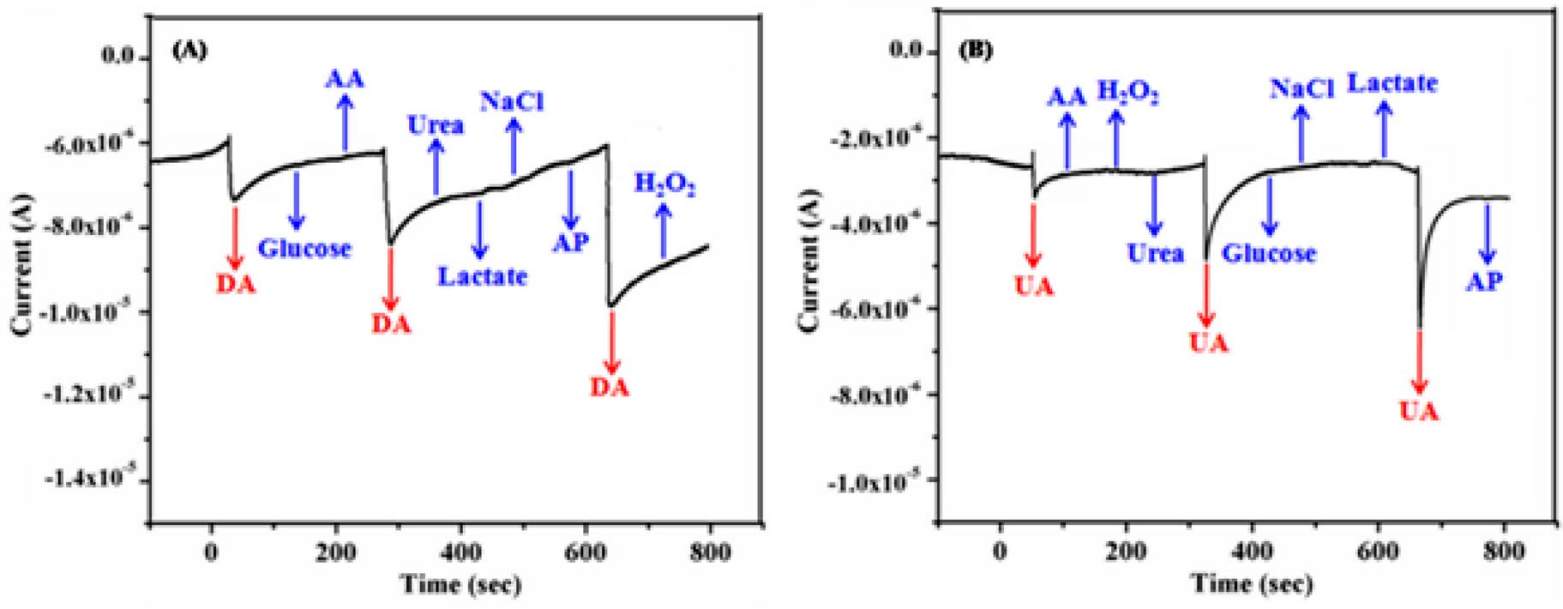
Amperometric (i–t) response of γ-Fe_2_O_3_ NWs modified GCE with the successive addition of 0.3 mM DA (**A**) and 0.3 mM UA (**B**) in presence of 3 mM (tenfold) concentrations of other potential interferences in PBS (pH—7.4) solution at an applied potential of + 0.25 V vs. SCE for DA and + 0.35 V vs. SCE for UA respectively.

Selectivity of the proposed electrochemical sensor towards the detection of DA and UA using γ-Fe_2_O_3_ NWs modified GCEs is examined in presence of several other possible interference species such as glucose, ascorbic acid, hydrogen peroxide (H_2_O_2_), acetaminophenon (AP), urea and sodium chloride. These studies are performed by monitoring amperometric responses of DA (A) and UA (B) in presence of the aforementioned species and the corresponding results are shown in Fig. 16A, B. The concentration values of these interfering species were kept at 10 times higher than the target analyte (0.3 mM DA and 0.3 mM UA) in PBS (pH—7.4) solution. It is clear that no obvious interference is observed, demonstrating the good selectivity of as-fabricated γ-Fe_2_O_3_ NWs coated GCE. From these results, it is understood that tenfold physiological and 100-fold common ion interference concentrations did not significantly affect the detection of DA and UA; manifesting a higher selectivity of the proposed biosensor^78–81^. These results vividly suggest that γ-Fe_2_O_3_ NWs modified GCE is highly selective towards DA and UA sensing when compared to the aforementioned interfering compounds.

### Stability of γ-Fe_2_O_3_ NWs/GCE

**Figure 17:**
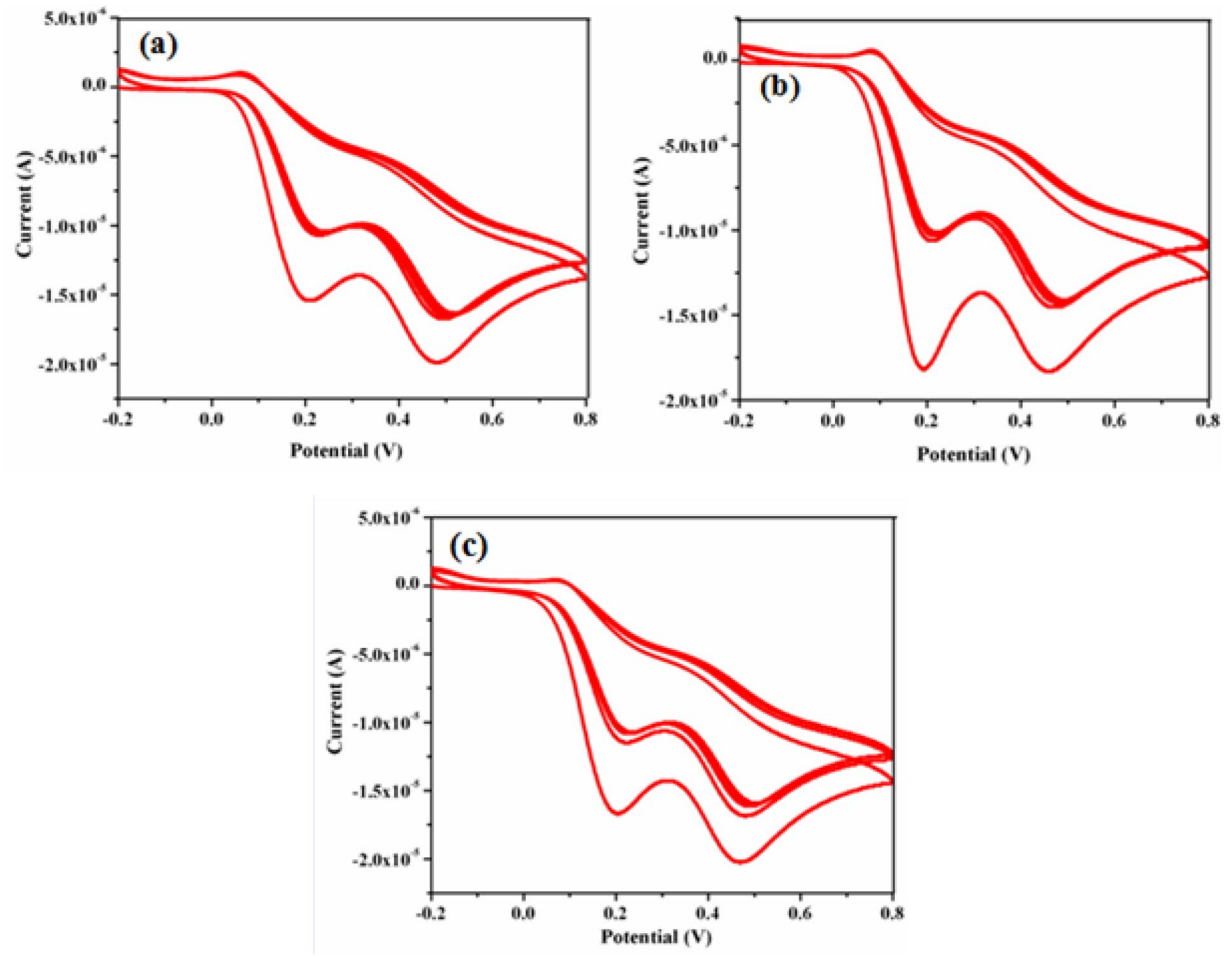
CVs of γ-Fe_2_O_3_ NWs/GCE recorded for 50 cycles in presence of 0.2 mM DA and UA for different time intervals of (**a**) 10 days, (**b**) 20 days and (**c**) 30 days respectively at a scan rate of 50 mV s^−1^.

Finally to prove the stability of γ-Fe_2_O_3_ NWs/GCE towards the electrochemical sensing performance, CV curves were recorded for different time intervals viz., 10, 20 and 30 days towards 0.2 mM DA and UA at a scan rate of 50 mV s^−1^. These studies were carried out by recording CV for 50 cycles by monitoring the change in peak current values and the corresponding CVs are shown in Fig. 17a–c. It can be noted from these CVs that mere a 2% decrease in peak current value was observed even after 50 cycles, indicating a good stability of the electrode.

Thus, these modified electrodes are successfully used for the simultaneous determination of DA and UA with excellent sensitivity and selectivity. Finally, from these electrochemical results, it can be concluded that γ-Fe_2_O_3_ nanostructures modified GCEs can act as a suitable redox mediator and shows potentially efficient electrocatalytic activity for the simultaneous determination of potential biomarkers such as DA and UA. Also, it overcomes the interest of using other film modified GCEs and found as a novel and economically viable one for the simultaneous determination of DA and UA. This study constitutes a simple and versatile protocol which can be used in an effective way to develop electrochemical sensors and biosensors.

## Experimental section

### Chemicals

Reagent grades of iron (III) chloride hexahydrate (FeCl_3_.6H_2_O), iron (II) sulphate heptahydrate (FeSO_4_·7H_2_O), sodium hydroxide (NaOH), uric acid and dopamine are purchased from E-Merck specialties products, India and were used as such without any further purification. Fenugreek seeds were purchased from the local market in Karaikudi, Tamilnadu, India. Double distilled water was used as a solvent to prepare the extract.

### Preparation of Fenugreek seed extract

Fenugreek (*Trigonella foenum-graecum*) is basically a spice crop belongs to the family of *Fabaceae*. It is a semi-arid plant and cultivated worldwide. Generally their pods contain 10 − 20 seeds that are cuboid in shape and yellow to amber in colour. They are mainly used to prepare extracts or powders for cuisines and for medicinal applications. For the preparation of extract, about 10 g of Fenugreek seeds were washed several times with distilled water to remove the surface impurities and then immersed in 50 mL of distilled water for about 12 h at room temperature. These seeds were grinded well and the extract was filtered using filter paper (Whatmann grade no. 10) and then centrifuged at 8,000 rpm for 15 min. Finally, the supernatant was separated and stored at 4 °C for further use. FTIR spectroscopy is used for the analysis of purity of such extract. In a typical experimental procedure, about 0.9, 1.8 and 4.5 mL of FS extracts were used to prepare 2%, 4% and 10% (v/v %) of FS utilized for the synthesis of various nanostructures of iron oxide.

### Synthesis of iron oxide nanostructures

Different nanostructures of iron oxide were prepared by an aqueous co-precipitation method using FeCl_3_·6H_2_O and FeSO_4_·7H_2_O as the primary sources of iron. In a systematic experimental procedure, 30 mL of 0.1 M FeCl_3_·6H_2_O solution was mixed with 15 mL of 0.1 M FeSO_4_.7H_2_O solution in a clean three-necked round bottom flask^25^. Appropriate amounts of saponin rich bio-surfactant, Fenugreek seed extracts were added into the above reaction mixture and allowed to react for an hour. After that, 10 M NaOH aqueous solution was slowly added into the above solution under vigorous stirring to bring the pH upto13 and the stirring was continued for another 2 h for the completion of reaction. Interestingly the colour of this particular solution turned from orange to black, consequently a reddish-brown precipitate was formed. The resulting solid product was removed; washed very well with double distilled water and dried at room temperature in atmospheric air. Following the above procedure, the experiments were carried out under various volume percentage values such as 2%, 4% and 10% of FS extract in the pre-fixed iron precursor’s ratio and also at different time intervals. This procedure yields different nanostructures of iron oxide as discussed in the results and discussion part.

### Characterization of iron oxide nanostructures

Formation of different nanostructures of synthesized γ-Fe_2_O_3_ and their structural and morphological characteristics were examined initially using field emission scanning electron microscope (FESEM, Hitachi MODEL S-4800)^18,25^. Similarly, transmission electron microscope (TEM) images were obtained using JEOL TEM 2010 microscope operated at 200 kV^18,25^. X-ray diffraction studies (XPERT-PRO with Cu Kα radiation [λ = 0.154060 nm], PANlytical X’Pert Pro-diffractometer) were carried out to evaluate the crystalline structure, phases and phase purity of the resultant iron oxide samples^25,39–42^. Fourier transform infrared spectrometer (FTIR, Nicolet 5700) was used to analyze the surface characteristics and the presence of chemical functionalities in the synthesized γ-Fe_2_O_3_ nanostructures. X-ray photoelectron spectroscopy (XPS) was recorded for different nanostructures of γ-Fe_2_O_3_ using Kratos ASIS-HS instrument equipped with a standard monochromatic source (Al K_α_) operated at 150 W (15 kV, 10 mA) to confirm the presence of elements along with their oxidation states and nature of the formed products^18,25^. Finally the magnetization hysteresis loops of different nanostructures were also characterized using a Lake model 7,300 vibrating sample magnetometer (VSM).

### Fabrication of sensor matrix using iron oxide nanostructures modified GCE

Synthesized γ-Fe_2_O_3_ nanostructures by employing different weight % of FS extracts were utilized further for the electrochemical detection of DA and UA by modifying GCEs with these materials. Prior to modification, GCE was polished to a mirror-like surface with progressively decreasing 1 μm, 0.3 μm and 0.05 μm alumina slurries and rinsed with double distilled water thoroughly between each polishing step^25,49,50^. Then it was washed successively with double distilled water in an ultrasonic bath and dried in air at room temperature. About 1 mg of a particular nanostructure of γ-Fe_2_O_3_ was dispersed in 3 mL of ethanol under ultrasonication. Subsequently γ-Fe_2_O_3_ modified GCEs were obtained by drop casting 5 μL of these suspensions onto the surface of pre-cleaned GCE^49,50^. Finally the modified GCE was activated in phosphate buffer solution (PBS) having pH 7.4 by successive cyclic scans between –0.2 and + 0.8 V^54–60^. Before and after each experiment, the modified GCE was washed well with distilled water and reactivated in PBS as mentioned above.

### Electrochemical sensing of DA and UA using Iron oxide Modified GCEs

Electrochemical sensing studies of DA and UA were performed using different nanostructures of γ-Fe_2_O_3_ modified GCEs and bare GCE without any coating (for comparison) in an aqueous solution consisting of 0.5 mM DA and UA. N_2_ gas was purged into the freshly prepared DA and UA solutions for about 5 min to eliminate the dissolved oxygen and overflowed to avoid the atmospheric oxygen interference during the electrochemical oxidation of target analyte^49,50,62–64^. All the electrochemical measurements such as cyclic voltammetry (CV), differential pulse voltammetry (DPV), and chronoamperometry (CA) were carried out using CHI 6131D Electrochemical Impedance Analyzer procured from USA using either the modified GCE or bare GCE as a working electrode, a platinum wire was used as a counter electrode, and saturated calomel electrode (SCE) was used as the reference electrode respectively^18,25,62–64,68^. Other details and necessary parameters were mentioned in the respective diagram provided in the results and discussion part.

## Conclusions

In conclusion, a distinct type of preparation of zero dimensional and 1D γ-Fe_2_O_3_ nanostructures with tunable sizes and shapes using fenugreek seeds extract as a new bio-surfactant has been reported. By careful tuning of the reaction parameters, pure phase of cubic spinel, superparamagnetic structures of γ-Fe_2_O_3_ NGs, NWs and NSs have been successfully synthesized using a simple one-pot process. XRD, XPS and FTIR results confirmed that the synthesized nanostructures posses pure crystalline phase of cubic spinel γ-Fe_2_O_3_. FESEM and TEM analyses showed that the obtained morphologies are in 0D and 1D iron oxide nanostructures with an average particle size in the range of 12 nm (0D) and width of 230–250 nm (1D) respectively. Furthermore, γ-Fe_2_O_3_ modified GCE shows efficient electrocatalytic activity for the simultaneous determination of DA and UA by significantly decreasing their oxidation overpotential values and enhancing the peak current values when compared to bare GCE without any modification. The proposed electrode is very easy to fabricate and eco-friendly for the simultaneous determination of biomolecules like DA and UA. A large value of peak separation observed between DA and UA also facilitates the simultaneous determination using γ-Fe_2_O_3_ coated GCE. Among the different nanostructures of γ-Fe_2_O_3_ studied in this work, NWs modified GCE showed an excellent selectivity and higher sensitivity for DA and UA detection, along with higher current response when compared to other nanostructures. It is also found that the other potentially interfering species showed negligible interference towards selective detection of DA and UA revealing a good selectivity of γ-Fe_2_O_3_. The relatively smaller sized unique nanostructures, higher surface area and the presence of surface hydroxyl groups are responsible for the enhanced electrocatalytic activity of γ-Fe_2_O_3_ NWs modified GCE. Many outstanding advantages, like appreciable sensitivity, wide linear concentration range, lower detection limit and selectivity of the modified electrodes confirmed that γ-Fe_2_O_3_ NWs possesses an excellent analytical performance by making them a promising candidate for electrochemical sensing applications. Our investigation provides an environmentally benign synthetic route for the preparation of iron oxide nanostructures, and the proposed method is eco-friendly, facile and novel that could be used for large scale production.

## Supplementary information

Supplementary information.
