# Supplementary material for: Bio-assisted preparation of efficiently architectured nanostructures of γ-Fe2O3 as a molecular recognition platform for simultaneous detection of biomarkers
Source: Sci Rep. 2020 Sep 15;10:15071. doi: 10.1038/s41598-020-71934-7 (PMC7493908; doi:10.1038/s41598-020-71934-7)
Supplement: Supplementary file 1 — Supplementary information. [file 41598_2020_71934_MOESM1_ESM.pdf]

Electronic Supporting Information

Bio-assisted Preparation of Efficiently Architectured Nanostructures of γ-Fe_2O_3 as a Molecular Recognition Platform for Simultaneous Detection of Biomarkers

Sasikala Sundar[a] and V. Ganesh[a,b,*]

[a] Electrodics and Electrocatalysis (EEC) Division, CSIR – Central Electrochemical Research Institute (CSIR – CECRI), Karaikudi – 630003, Tamilnadu, India.

[b] Academy of Scientific and Innovative Research (AcSIR), Ghaziabad – 201002, India.

* Corresponding Author's E-mail: vganesh@cecri.res.in (or) ganelectro@gmail.com

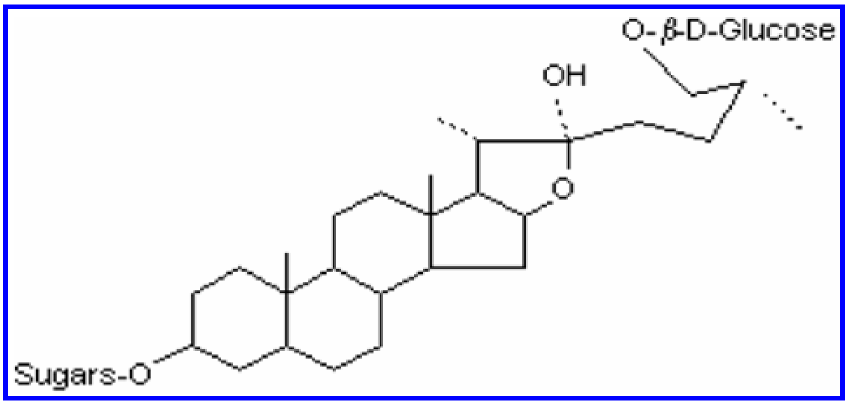

Fig. S1 Chemical structure of Furostanol saponin (FS) present in the extract of Fenugreek seeds.

Fenugreek seeds contain saponins in the form of furostanol saponins and it may be defined as bidesmosidic saponins that have two sugar chains, with one bonded at C3 and one attached through an ether linkage at C26 with a D-glucose unit [1].

PDF # 895892, Wavelength = 1.54060 (A)

89-5892 Quality: C

CAS Number:

Molecular Weight: 1692.42
Volume[CD]: 581.28
Dx: 4.835 Dm:

Sys: Cubic
Lattice: Primitive
S.G.: P4332 (212)
Cell Parameters:
a 8.345 b c
α β γ

I/Icor: 4.39
Rad: CuKa1
Lambda: 1.54060
Filter:
d-sp: calculated

ICSD #: 087119

Mineral Name:
Maghemite

γ-Fe21.16 O31.92
Iron Oxide
Ref: Calculated from ICSD using POWD-12++
Ref: Shin, H.-S., Yoop Hakhoechi, 35, 1113 (1998)

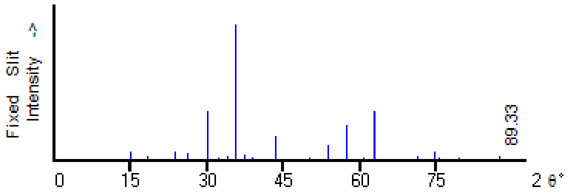

2θ	Int-f	h	k	l	2θ	Int-f	h	k	l	2θ	Int-f	h	k	l
15.001	58	1	1	0	51.306	1	3	3	2	73.477	1	5	4	1
18.398	29	1	1	1	53.766	103	4	2	2	74.493	63	5	3	3
23.821	62	2	1	0	54.967	4	4	3	0	75.504	15	6	2	2
26.133	54	2	1	1	56.151	5	5	1	0	76.510	3	5	4	2
30.266	362	2	2	0	57.319	260	5	1	1	77.511	1	6	3	1
32.150	12	2	2	1	59.609	9	5	2	0	79.504	16	4	4	4
33.941	25	3	1	0	60.735	15	5	2	1	80.496	3	6	3	2
35.651	999 *	3	1	1	62.949	353	4	4	0	81.484	1	7	1	0
37.294	35	2	2	2	64.040	5	4	4	1	82.470	1	5	5	1
38.876	15	3	2	0	65.121	7	5	3	0	84.435	1	7	2	0
40.407	7	3	2	1	66.193	2	5	3	1	85.415	4	5	5	2
43.332	173	4	0	0	67.255	1	4	4	2	87.372	29	6	4	2
44.737	2	4	1	0	68.310	4	6	1	0	88.349	1	7	2	2
46.107	4	3	3	0	69.357	6	5	3	2	89.325	3	7	3	0
47.447	1	3	3	1	71.430	33	6	2	0					
50.044	18	4	2	1	72.456	4	6	2	1					

Fig. S2 Standard XRD data – JCPDS No. 89-5892 of γ-Fe_2O_3 (Maghemite).

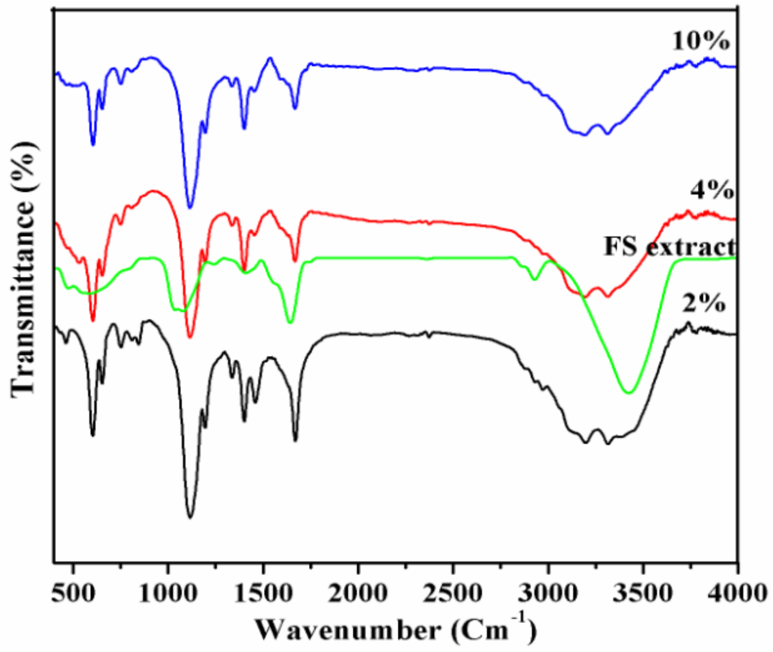

Fig. S3 FTIR spectra recorded for γ-Fe_2O_3 nanostructures synthesized using different volume % (2, 4 and 10%) of FS extract for 30 minutes time duration and for comparison FTIR spectrum of pure FS is also shown.

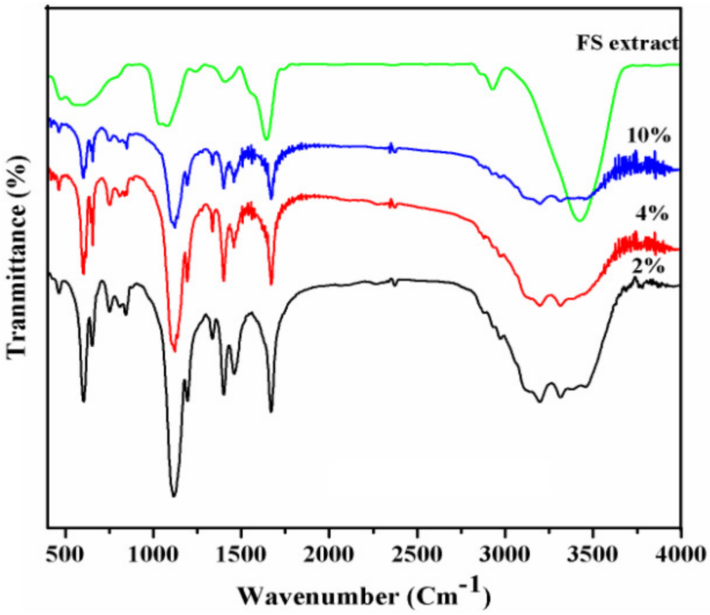

Fig. S4 FTIR spectra recorded for γ-Fe_2O_3 nanostructures synthesized using different volume % (2%, 4% and 10%) of FS extract for 3 h time duration and for comparison FTIR spectrum of pure FS is also shown.

Figures S3 and **S4** illustrate the FTIR spectra of different samples (NGs, NWs and NSs of γ-Fe_2O_3) synthesized using various volume % of FS and pure FS extract for 30 minutes and for 3 h time duration respectively. FTIR spectra of all the iron oxide nanostructures display a characteristic –OH stretching (ν OH) and –H-O-H bending (δ OH) and vibrational bands in the region from 3100 cm^{-1} to 3400 cm^{-1} respectively. These observations could be attributed to the stretching vibrations of adsorbed moisture and surface hydroxyl groups present on the surface of iron oxide.

From these FTIR spectra, it can be seen that the appearance of transmittance peak at 604 cm^{-1} corresponds to the stretching vibration of tetrahedral iron atoms (ν Fe-O) present in the synthesized iron oxide samples [2]. The sharp peaks centered at 1100 cm^{-1} in all these spectra could be assigned to (δ C-O-H) stretching vibration of saponin molecule present in the FS extract and the peak at ~1620 cm^{-1} represents pure –CH_2 group vibration of sugar moieties found in FS [3,4]. Similarly the band appeared at ~1370 cm^{-1} could be attributed to the deformation vibration of C-H bond of alkane present in the FS extract. FTIR spectrum of FS extract suggests the purity of FS extracted from Fenugreek seeds. Further these results confirm the presence of functional groups of FS extract and also suggested that the synthesized nanostructures are in the pure phase of γ-Fe_2O_3, very well correlated with XRD and XPS studies.

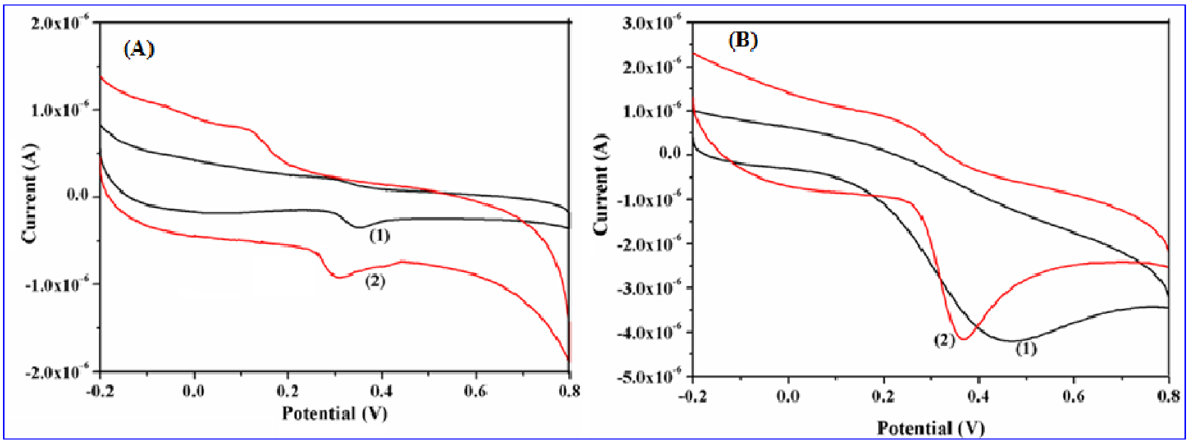

Fig. S5 Cyclic voltammetric responses obtained using (1) bare GCE and (2) γ-Fe_2O_3 NGs modified GCEs in PBS (pH = 7.4) at a fixed scan rate of 50 mV s^{-1} for (A) 0.5 mM DA and (B) 0.5 mM UA respectively.

Fig. S5 shows the CV responses observed for the electrocatalytic activity of DA (A) and UA (B) at γ-Fe_2O_3 NGs modified GCE and bare GCE without any coating. It can be seen from these CVs that although NGs modified GCE displayed a better response than bare GCE, it is found to be much lower when compared to that of both NWs and NSs of γ-Fe_2O_3 modified GCE. Hence the electrochemical sensing experiments for the detection of DA and UA were subsequently carried out with NWs and NSs of γ-Fe_2O_3.

Table S1. Cyclic voltammetric parameters determined for individual electrocatalytic oxidation of DA and UA at bare GCE, γ-Fe_2O_3 NSs and γ-Fe_2O_3 NWs modified GCEs.

	DA Sensor		UA Sensor	
**Electrode Material**	**Current (A)**	**Voltage (mV)**	**Current (A)**	**Voltage (mV)**
**Bare GCE**	3.67×10^{-6}	300	5.61×10^{-6}	490
**γ-Fe_2O_3 nanospheres / GCE**	2.54×10^{-5}	290	8.75×10^{-6}	350
**γ-Fe_2O_3 nanowires / GCE**	3.72×10^{-5}	250	1.42×10^{-5}	330
**γ-Fe_2O_3 nanograsses / GCE**	9.89×10^{-7}	300	4.19×10^{-6}	370

Table S2. Cyclic voltammetric parameters deduced for the simultaneous electrocatalytic oxidation of DA and UA at bare GCE, γ-Fe_2O_3 NSs and γ-Fe_2O_3 NWs modified GCEs.

	DA Sensor		UA Sensor	
**Electrode Material**	**Current (A)**	**Voltage (mV)**	**Current (A)**	**Voltage (mV)**
**Bare GCE**	--	--	--	--
**γ-Fe_2O_3 nanospheres / GCE**	1.37×10^{-5}	220	1.68×10^{-5}	450
**γ-Fe_2O_3 nanowires / GCE**	1.89×10^{-5}	200	2.10×10^{-5}	430

References

[1] M. Majeed, L. Prakash, Fenusterols® – Product Insight Paper, Sabinsa Corporation, 2007, pp. 1–8.

[2] D. Predoi, A study on iron oxide nanoparticles coated with dextrin obtained by co-precipitation, Dig. J. Nanomater. Biostruct. 2 (2007) 169–173.

[3] J. Hradil, A. Pisarov, M. Babic, D. Horak, Dextran-modified iron oxide nanoparticles, China Particuology 5 (2007) 162–168.

[4] C. Singh, M. A. Ali, G. Sumana, Green synthesis of graphene based biomaterial using fenugreek seeds for lipid detection, ACS Sustainable Chem. Eng. 4 (2016) 871–880.
